# Glial maturation factor-β deficiency prevents oestrogen deficiency-induced bone loss by remodelling the actin network to suppress adipogenesis of bone marrow mesenchymal stem cells

**DOI:** 10.1038/s41419-024-07234-z

**Published:** 2024-11-14

**Authors:** Jun Xu, Zhongyue Huang, Si Shi, Jiangni Xia, Guangnan Chen, Kaifeng Zhou, Yiming Zhang, Chong Bian, Yuqin Shen, Xiaofan Yin, Lixia Lu, Huijie Gu

**Affiliations:** 1 Department of Orthopedics, Minhang Hospital, Fudan University, Shanghai, PR China; 2 Department of Rehabilitation, Tongji Hospital Affiliated to Tongji University, Tongji University School of medicine, Shanghai, PR China; 3 Department of Biochemistry and Molecular Biology, Tongji University School of medicine, Shanghai, PR China

**Keywords:** Osteoporosis, Mesenchymal stem cells

## Abstract

An imbalance between the adipogenesis and osteogenesis of bone marrow mesenchymal stem cells (BMSCs) is considered the basic pathogenesis of osteoporosis. Although actin cytoskeleton remodelling plays a crucial role in the differentiation of BMSCs, the role of actin cytoskeleton remodelling in the adipogenesis of BMSCs and postmenopausal osteoporosis (PMOP) has remained elusive. Glia maturation factor-beta (GMFB) has a unique role in remodelling the polymerization/depolymerization cycles of actin. We observed that GMFB expression was increased in bone tissue from both ovariectomized (OVX) rats and PMOP patients. GMFB knockout inhibited the accumulation of bone marrow adipocytes and increased bone mass in the OVX rat model. The inhibition of adipocyte differentiation in GMFB knockout BMSCs was mediated via actin cytoskeleton remodelling and the Ca^2+^-calcineurin-NFATc2 axis. Furthermore, we found that GMFB shRNA treatment in vivo had favourable effects on osteoporosis induced by OVX. Together, these findings suggest a pathological association of the GMFB with PMOP and highlight the potential of the GMFB as a therapeutic target for osteoporosis patients.

## Introduction

Osteoporosis (OP), particularly postmenopausal osteoporosis (PMOP), is characterized by compromised bone formation and increased adipocytes in the bone marrow [1–3]. Bone marrow mesenchymal stem cells (BMSCs) are common progenitors of osteoblasts and adipocytes. BMSCs preferentially differentiate into adipocytes during PMOP [4, 5]. However, the molecular mechanisms by which BMSCs favour adipogenesis to osteogenesis remain unclear.

Actin cytoskeleton remodelling is known to play a crucial role in the differentiation of BMSCs into adipogenic and osteogenic lineages [6]. Actin polymerization is observed in osteogenesis, whereas actin depolymerization is observed during adipogenesis [7, 8]. Disruption of actin stress fibres is an essential element for adipogenesis and precedes the induction of PPARγ expression during adipocyte differentiation [9]. Stabilizing actin filaments in BMSCs reduces adipocyte differentiation, whereas disruption of the actin cytoskeleton enhances adipocyte differentiation [10]. Inhibition of the Arp2/3 complex, which forms the secondary actin filament branch, results in unbranched actin polymers and strongly promotes adipogenesis [11]. However, the role of actin cytoskeleton remodelling in the adipogenesis of BMSCs and PMOP has remained elusive.

Glia maturation factor-beta (GMFB) is a member of the actin regulatory protein ADF-H family [12]. GMFB has a unique role in remodelling the polymerization/depolymerization cycles of actin. GMFB binds the Arp2/3 complex and promotes the debranching of actin filament networks [13]. Moreover, GMFB is considered a growth/differentiation factor and a regulator of intracellular pressure-related signals [14, 15]. Ectopic overexpression of GMFB in nonbrain cells can lead to apoptosis and premature ageing through the susceptibility of cells to oxidative stress [16]. GMFB-deficient astrocytes exhibit increased resistance to oxidative stress [17]. GMFB is upregulated after partial hepatectomy and plays an active role in the process of liver regeneration [18]. However, the role of GMFB in the adipogenesis of BMSCs and the occurrence of osteoporosis has not been reported in detail.

In the present study, we first explored the role of GMFB in PMOP and adipogenesis of BMSCs in vivo and in vitro. GMFB expression was increased in bone tissue from both OVX rats and PMOP patients. GMFB knockout inhibited the accumulation of bone marrow adipocytes and increased bone mass in the OVX rat model. GMFB knockout inhibited the adipocyte differentiation of BMSCs via actin cytoskeleton remodelling and the Ca^2+^-calcineurin-NFATc2 axis. Furthermore, we found that GMFB shRNA treatment in vivo had protective effects on osteoporosis induced by OVX.

## Methods and materials

### Preparation of PMOP patient-derived bone samples

This study was approved by the Ethics Committee of Minhang Hospital of Fudan University. A total of 19 patients who underwent spine-related surgeries at Shanghai Minhang Hospital of Fudan University were enrolled in this study. All patients signed written informed consent. The inclusion criteria are listed in Table S1. The vertebral bone samples were collected as described previously [19].

### Generation of GMFB knockout rats

In this study, female Sprague‒Dawley rats (6–12 weeks old) were purchased from Slack Laboratory Animal Company, Shanghai. GMFB knockout rats were generated via the CRISPR‒Cas9 system. PCR analyses of genomic DNA were used to identify the genotype of the transgenic rats. All the studies performed were approved by the Institutional Animal Care and Use Committee of Fudan University.

### Preparation of the OVX rat model

In this study, 8-week-old SD female GMFB knockout and wild-type rats were selected for ovariectomy. There was no significant difference in the initial body weight of the rats. A sham surgery or an ovariectomy was performed on the rats after anaesthesia via pentobarbital sodium (body weight of 50 mg/kg, i.p.). The ovariectomy was performed according to a previous study [20], and the rats were then randomly divided into 4 groups, each with eight mice: I, sham-operated wild-type rats; II, sham-operated GMFB KO rats; III, OVX wild-type rats; IV, OVX GMFB KO rats.

### BMD measurement

Eight weeks after OVX, the BMD of the femur was measured via dual-energy X-ray absorptiometry (DXA; GE Healthcare, Madison, WI, USA).

Human BMD was measured using a DXA fan-beam bone densitometer (Hologic QDR 4500 A; Hologic) at the lumbar spine, total hip, and femoral neck 1–2 days before surgery. The X-ray absorptiometer was calibrated, and reference values were obtained according to a previous study [21]. The regions of severe scoliosis, fracture, and sites of operation were excluded from the BMD measurements.

### Micro-CT analysis

The harvested bone tissues were fixed in 4% polyoxymethylene for 2 days and then scanned and analysed using micro-computed tomography (micro-CT; Bruker Corp., Billerica, MA, USA). The scanner was set at a spatial resolution of 8 μm per pixel, a voltage of 55 kV, and a current of 114 mA. The proximal tibia was used to determine the ratio of the trabecular bone volume/tissue volume (BV/TV), the trabecular thickness (Tb.Th), the trabecular number (Tb.N), and the trabecular separation (Tb.Sp). The region of interest (ROI) of the cortical bone was 30% of the length of the middle tibia, and the bone volume (BV/TV) and cortical thickness (Ct. Th) were calculated.

### Histomorphometric assay and toluidine blue staining

Histomorphometric analysis was performed as previously described [22]. The rats were injected with calcein (15 mg/kg) 8 days and 2 days before euthanasia. Tibias were soaked in 70% ethanol and embedded in methyl methacrylate. The samples were sectioned into 5 μm sections for dynamic histomorphometry. The mineral apposition rate (MAR) and bone formation rate (BFR/BS) were calculated for each sample. In addition, the bone sections were processed for toluidine blue staining. The sample area selected for bone analysis was a 1 mm^2^ area within the metaphyseal secondary spongiosa, originating 1 mm below the growth plate.

### Immunohistochemical and immunofluorescence

Immunohistochemical staining was performed via a standard protocol as previously described [23]. The bone sections were digested with 0.05% trypsin at 37 °C for 15 min for antigen retrieval and subsequently incubated with primary antibodies against osteocalcin (Abcam, ab83976) and GMFB (Proteintech, 10690-1-AP) at 4 °C overnight. Then, immunoactivity was detected via an HRP-streptavidin detection system (DAKO, Carpinteria, CA, USA).

For immunostaining, the sections were incubated with primary antibodies against GMFB (Proteintech, 10690-1-AP), CD105 (R&D Systems, AF6440), and NFATc2 (Cell Signaling Technology, 4389S). Nuclei were counterstained with DAPI (Sigma, D9542). The sections were then observed under a fluorescence microscope (Olympus, Japan).

### Primary BMSC isolation and culture

We flushed the bone marrow of the tibias and femurs to isolate primary rat BMSCs. The cells were cultured in the following growth medium (GM): low-glucose Dulbecco’s modified Eagle’s medium (LG-DMEM) (Gibco, 11885084) supplemented with 10% foetal bovine serum (FBS) (Gibco, A5670701), 2 mM glutamine (Sigma, 1294808), 100 mg/mL streptomycin (Sigma, P4458), and 100 U/mL penicillin (Sigma, P4458). The medium was changed twice a week.

### Adipogenic differentiation assay

To induce adipogenic differentiation, the following BMSCs were cultured in adipogenic medium (AM): LG-DMEM containing 10% foetal bovine serum (FBS) (Gibco, 11885084), 2 mM glutamine (Sigma, 1294808), 100 mg/mL streptomycin (Sigma, P4458), 100 U/mL penicillin (Sigma, P4458), 0.5 mM isobutylmethylxanthine (IBMX) (Sigma, I5879), 1 μM Dex (Sigma, D4902), 10 μM insulin (Sigma, 91077 C), and 200 μM indomethacin (Sigma, I7378) for 7 days. Lipid formation was detected by Oil Red O (Sigma, O0625) staining.

#### Phalloidin staining of F-actin

To visualize F-actin in cells, cultured BMSCs were fixed and stained with phalloidin (200 nM, 40734ES80; Yeasen, Shanghai, China). The cell nucleus was then stained with DAPI. The mean F-actin intensity in each group was measured using ImageJ software (USA).

#### Calcium imaging

WT and KO BMSCs were incubated with GM or AM for 30 min and then loaded with Fluo-4 AM (5 μM, Beyotime, S1060) for approximately 30 min. Calcium imaging was performed with a fluorescence microscope system (Olympus, Japan). Changes in the intracellular Ca^2+^ concentration were measured from fluorescence images of Fluo-4 AM (excitation at 488 nm, emission at 520 ± 20 nm).

### Western blotting analysis

The cells were lysed using RIPA buffer supplemented with proteinase and phosphatase inhibitors. Total lysates were separated by sodium dodecyl sulfate‒polyacrylamide gel electrophoresis (SDS‒PAGE) and transferred onto a polyvinylidene difluoride (PVDF) membrane. The membrane was blocked and then incubated with primary antibodies at 4 °C overnight. The primary antibodies used were as follows: GMFB (10690-1-AP, 1:1000, Proteintech), β-actin (20536-1-AP, 1:2000, Proteintech), ERK (4695T, 1:1000, Cell Signaling Technology), phospho-ERK (4370T, 1:1000, Cell Signaling Technology), JNK (9252T, 1:1,000, Cell Signaling Technology), phospho-JNK (4668T, 1:1000, Cell Signaling Technology), P38 (8690T, 1:1000, Cell Signaling Technology), phospho-P38 (4511T, 1:1000, Cell Signaling Technology), β-catenin (8480T, 1:1000, Cell Signaling Technology), NFATc2 (4389S, 1:1000, Cell Signaling Technology), and Lamin-B1 (13435T, 1:1000, Cell Signaling Technology). The membrane was washed and visualized with appropriate horseradish peroxidase (HRP)-conjugated secondary antibodies (DAKO, Carpinteria, CA, USA). The corresponding bands were detected using an enhanced chemiluminescent (ECL) detection kit (36208ES60, Yeasen).

### Quantitative reverse transcription polymerase chain reaction

Total RNA was isolated from samples using TRIzol (10296010; Invitrogen, Carlsbad, CA, USA) and reverse transcribed into cDNA using PrimeScript RT polymerase (RR036A; TaKaRa, Kusatsu, Shiga, Japan). Real-time reverse transcriptase RT‒PCR was performed using a GeneAmp PCR System 9600 (PerkinElmer, Waltham, MA, USA). The primer sequences are listed in Table S2 and were synthesized by Sangon Biotech Co., Ltd. (Shanghai, China). Amplification reactions were set up in 25 μl reaction volumes containing SYBR Green Premix (FP205, Tiangen, Beijing, China), primers and cDNA.

### RNA sequencing

Total RNA was isolated from adipogenic differentiated BMSCs from WT and KO rats via TRIzol reagent. RNA library construction, sequencing and analysis were performed by Shanghai Oebiotech Co. Ltd. (Shanghai, China). GO and KEGG analyses were subsequently performed to determine the biological functions of these genes.

### Virus packaging and injection

The recombinant adeno-associated serotype 9 virus for gene delivery of the shRNA of the GMFB system (rAAV9-shRNA-Cherry, 5′-GCUUCAUUGUGUAUAGUUAUA-3′, 5′-UAACUAUACACAAUGAAGCGA-3′) was generated by Shanghai Taitool Bioscience Co., Ltd. (Shanghai, China). Clones were confirmed by DNA sequencing prior to use, and virus titres were determined by dot blot. An AAV viral titre of 5 × 10^12^ vector genomes/ml was used in the study. rAAV9-Cherry was used as a control. The rats received 20 μl of either rAAV9-shRNA-Cherry or rAAV9-Cherry via periosteal injection into the medullary cavity of the femur twice per month for 2 months.

### Statistical analysis

The data were statistically analysed using Prism 8 software, and the results are presented as the means ± standard deviations (SDs). Statistical analyses were conducted via one-way analysis of variance (ANOVA) or Student’s t-test. Correlation analysis was performed using Pearson product‒moment correlation. The enumeration data were compared via the chi-square test. *p* < 0.05 was considered statistically significant.

## Results

### GMFB expression is upregulated in PMOP

**Fig. 1:**
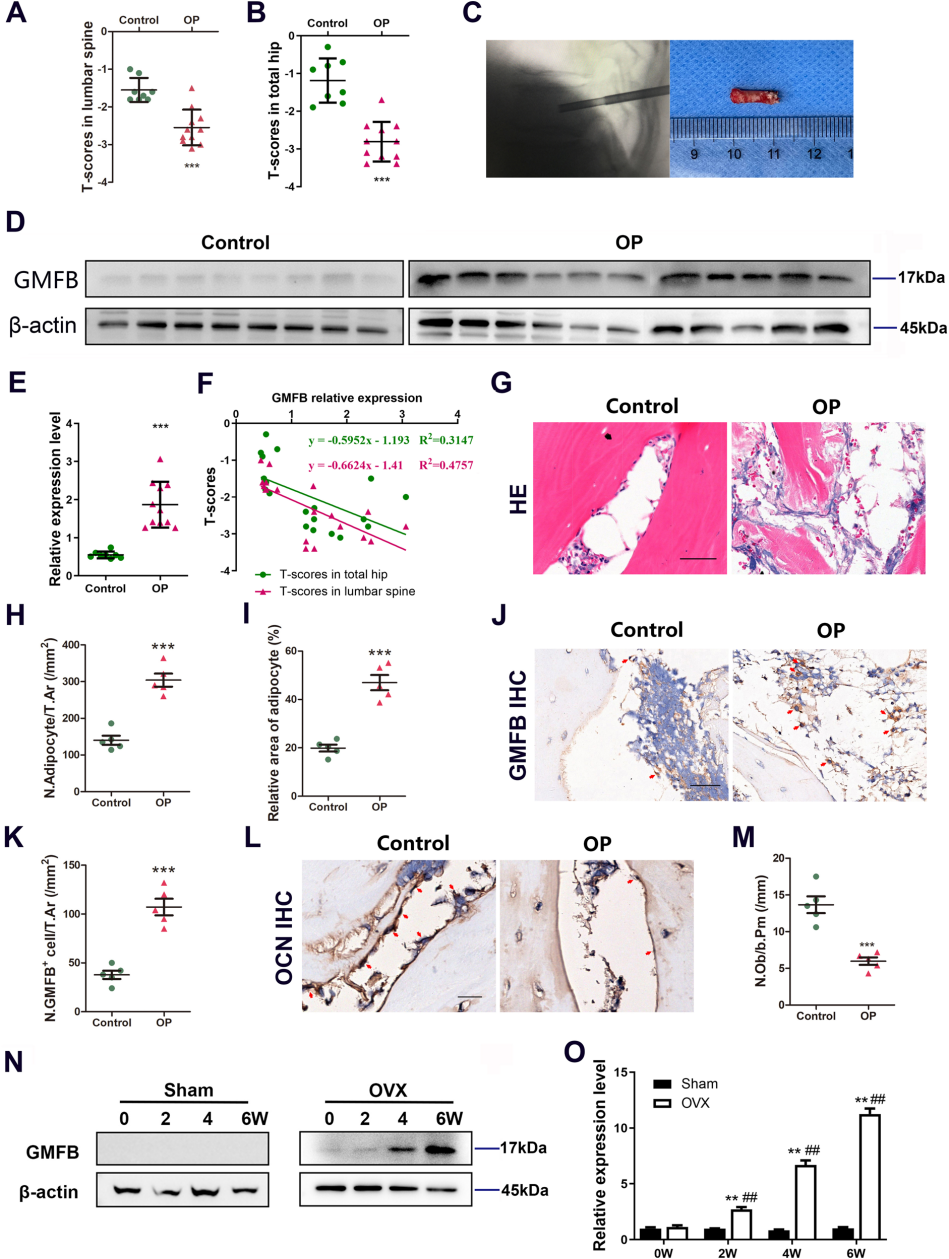
GMFB expression in bone extracts derived from PMOP patients and OVX rats. **A**, **B** T scores in lumber spine (**A**) and total hip (**B**) of the control group and the PMOP group. ***P < 0.001, unpaired Student’s t-test. **C** Preparation of patient-derived bone samples. **D**, **F** Western blot (**D**) and quantification (**E**) of GMFB in the bone tissues of the osteoporotic patients and healthy controls. Protein expression of GMFB is inversely correlated with T scores in the lumber spine (green) and total hip (red) of patients (**F**). Correlation formula was as follows: y = −0.5952x − 1.193 and y = −0.6624x − 1.41 (x: GMFB expression, y: T scores). **G**, **I** Representative images of H&E staining (**G**) and quantification of adipocyte number (**H**) and area (**I**) in bone samples from osteoporotic patients and healthy controls. (scale bars, 100 μm, n = 5). **J** and **K** Representative images of GMFB staining (**J**) and quantification of the number of GMFB^+^ cells (red arrows) in bone samples from osteoporotic patients and healthy controls (**K**) (scale bars, 50 μm, n = 5). **L** and **M** Representative images of osteocalcin (OCN) staining (**L**) and quantification of number of OCN^+^ cells (red arrows) in bone samples from osteoporotic patients and healthy controls (**M**) (scale bars, 50 μm, n = 5). **N** Western blot analysis of GMFB protein levels in bone tissue from the OVX and Sham rats after operation. **O** Quantification of band density in (**N**). Data represent the mean ± SEM of three independent experiments. Statistical analysis was performed using Student’s t-test. **p < 0.01 compared to the respective sham group. ##p < 0.01 compared to sham group at 0 weeks.

Nineteen patients were ultimately enrolled in this study. The clinical characteristics of the patients are shown in Table S3. The demographic data of the patients are presented in Table S4. The ages (in years) of the osteoporosis and control groups were 69.4 ± 7.6 and 64.9 ± 8.6, respectively. The heights (cm) of the osteoporosis and control groups were 159 ± 5 and 158 ± 4, respectively. There was no significant difference in age or height between the osteoporotic patients and the healthy controls. Weight values (63.9 ± 8.2 kg) in the healthy controls were greater than those (57.2 ± 7.2 kg) in the osteoporotic group; however, BMI values (25.4 ± 3.9 kg/m^2^) in the healthy controls were not significantly different from those (22.8 ± 2.7 kg/m^2^) in the osteoporotic group. The T scores of the lumber spine and total hip in osteoporosis patients (−2.81 ± 0.52 and −2.55 ± 0.47) were lower than those in controls (−1.55 ± 0.32 and −1.19 ± 0.59) (Fig. 1A, B).

The glucose and lipid metabolism of osteoporosis patients and healthy controls did not significantly differ (Fig. S1A–C). Our serological test results further confirmed that the included patients did not have endocrine diseases. There was no significant difference in the serum levels of thyroxine, parathyroid hormone, cortisol, or aldosterone between patients with osteoporosis and healthy controls (Fig. S1D–G). We excluded the impact of immune diseases on the research results by detecting anti-O antibodies and rheumatoid factors. There was no significant difference in anti-O antibodies or rheumatoid factors between osteoporotic patients and healthy controls (Fig. S1H, I). The levels of bone metabolic markers in the serum were determined. There was no significant difference in calcium, phosphorus, β-CTX or PINP between osteoporosis patients and healthy controls (Fig. S2A–D). The (25-HO) Vit D concentration in osteoporosis patients was lower than that in controls (−1.55 ± 0.32 and −1.19 ± 0.59) (Fig. S2E).

We also examined GMFB protein expression in the bone tissue of the included patients (Fig. 1C). GMFB protein expression was significantly greater in bone samples from osteoporotic patients than in those from healthy controls (Fig. 1D, E, Fig. S3). We analysed the T scores and GMFB protein levels in the included patients and found a negative correlation between T scores and GMFB protein expression (Fig. 1F). The HE staining results of the PMOP group revealed increased adipocyte accumulation in the bone tissue compared with that of the control group (Fig. 1G–I). Immunostaining of bone tissue sections revealed an increase in the number of GMFB-positive cells (Fig. 1J, K) and a decrease in the number of osteoblasts in osteoporotic patients compared with controls (Fig. 1L, M).

### Deletion of GMFB in rats prevents the oestrogen deficiency-induced increase in bone marrow adipose tissue

To elucidate the mechanism of postmenopausal osteoporosis, we allocated 8-week-old female rats to the sham group (subjected to sham surgery) or OVX group (subjected to ovariectomy). In OVX rats, HE staining revealed increased adipocyte accumulation in the tibia bone marrow, and older OVX rats had more adipocytes in the bone marrow (Fig. S4). To study the potential role of GMFB in bone formation and osteoporosis, we first examined the GMFB protein level in OVX rat bone tissues. Western blot analysis revealed that GMFB was expressed in the bone tissue of rats four and six weeks after OVX (Fig. 1N, O, Fig. S5). We subsequently generated GMFB knockout rats via the CRISPR/Cas9 technique (Fig. S6A). Western blot analysis and immunofluorescence staining of the tibia confirmed that the deletion of GMFB was successful (Fig. S6B, C). Compared with control littermates, GMFB knockout rats survived normally after birth and had no differences in size, weight, growth or fecundity. Eight-week-old GMFB knockout female rats and their control littermates were then subjected to OVX.

**Fig. 2:**
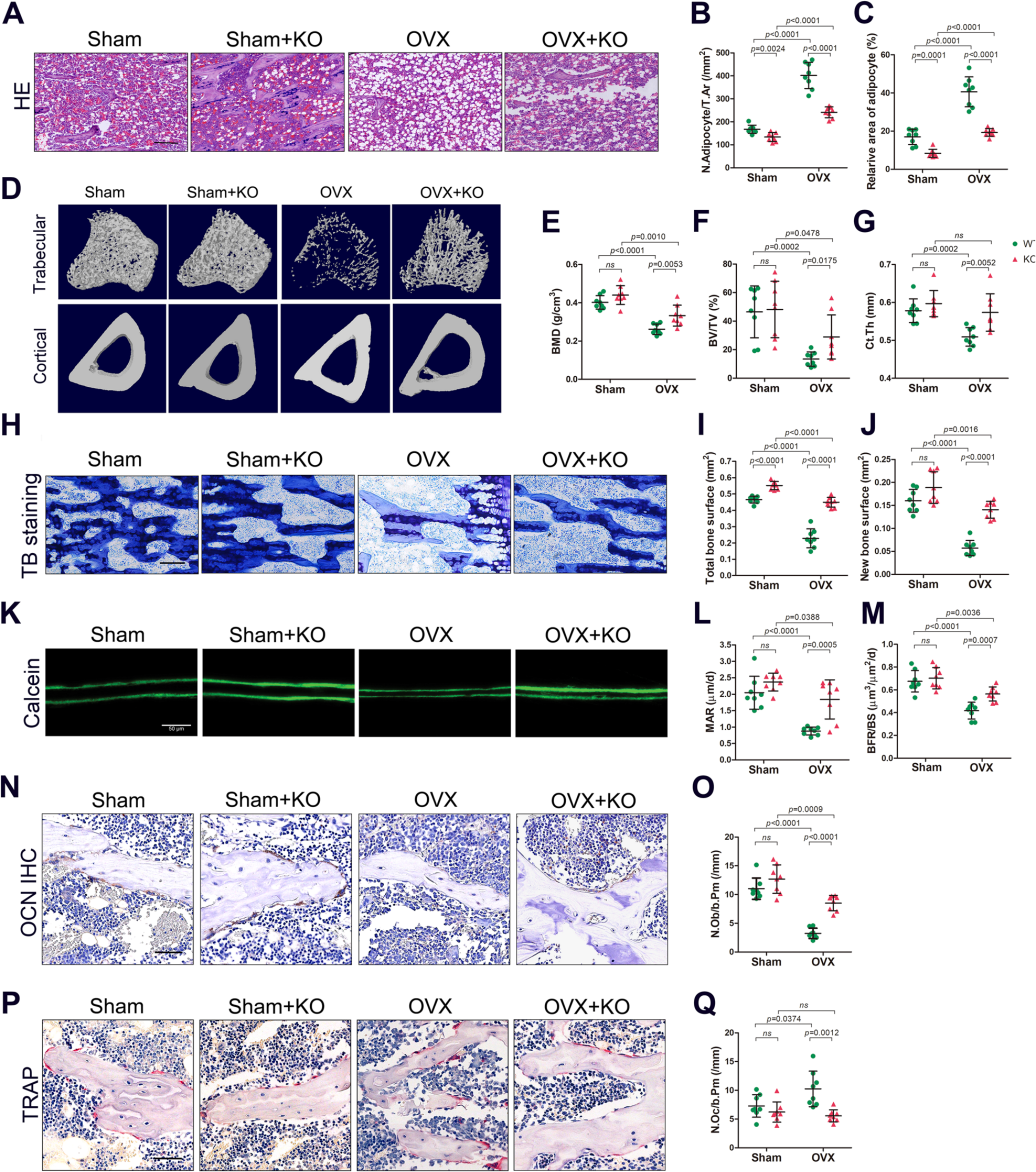
Gmfb KO decreased BMAT and attenuated the osteoporotic phenotype in OVX-induced OP in vivo. **A**–**C** Representative images of H&E staining (**A**) and quantification of adipocyte number (**B**) and area (**C**) in tibias from WT and GMFB KO female rats treated with either sham or OVX 8 weeks. (scale bars, 100 μm, n = 8). **D**–**G** Representative micro-CT images (**D**) and quantification of bone mineral density (BMD) (**E**), trabecular bone volume per tissue volume (Tb.BV/TV) (**F**), and cortical bone thickness (Ct.Th) (**G**) in tibias (n = 8). **H**–**J** Toluidine blue staining of the metaphyseal trabecular bone (**H**) and quantification of total bone surface (**I**) and new bone surface (**J**) in the tibias (scale bar, 100 μm, n = 8). **K**–**M** Representative images of calcein double-labelling (**K**) and analysis of mineral apposition rates (MARs) (**L**) and Bone formation rate per unit of bone surface (BFR/BS) (**M**) of trabecular bone in tibias (scale bars, 50 μm, n = 8). **N** and **O** Representative images of osteocalcin (OCN) staining (**N**) and quantification of number of OCN^+^ cells in tibias (**O**) (scale bars, 50 μm, n = 8). **P** and **Q** Representative images of tartrate-resistant acid phosphatase (TRAP) staining (**N**) and quantification of number of Trap^+^ cells in tibias (**O**) (scale bars, 50 μm, n = 8).

Eight weeks after OVX, the absolute weight and increasing weight of the OVX rats were significantly greater than those of the sham rats, whereas GMFB KO prevented the weight increase after OVX (Fig. S7A, B). HE staining of the tibias of the KO OVX rats revealed decreased adipocyte accumulation compared with that of the WT OVX rats (Fig. 2A). Both the number and the area of adipocytes in the bone marrow decreased in the KO OVX rats (Fig. 2B, C). However, there was no difference in fat vacuoles in the bone marrow cavity between WT sham rats and KO sham rats (Fig. 2A–C). These data suggest that GMFB regulates bone marrow adipose tissue accumulation in OVX rats.

### Deletion of GMFB in rats prevents oestrogen deficiency-induced osteoporosis

The micro-CT images revealed that the trabecular bone of the proximal tibia metaphysis in OVX rats decreased with increasing time, whereas GMFB KO prevented the reduction in trabecular bone in OVX rats (Fig. 2D and Fig. S7C). After 8 weeks, although GMFB KO rats did not exhibit significant changes in bone mass, micro-CT analysis revealed that the bone mineral density (BMD) and bone volume/tissue volume ratio (BV/TV) were significantly greater in KO OVX rats than in WT OVX rats (Fig. 2E, F). In addition, compared with WT OVX rats, GMFB KO OVX rats presented increased trabecular number (Tb.N), trabecular thickness (Tb.Th), and midshaft cortical thickness (Ct.Th) but decreased trabecular separation (Tb.Sp) and trabecular bone pattern factor (Tb.Pf) (Fig. S7D–G).

Toluidine blue staining further confirmed the high-bone mass phenotype of the GMFB KO OVX rats compared with the WT OVX rats (Fig. 2H–J). Moreover, the GMFB KO OVX rats had faster MAR and BFR, with more osteoblasts (N.Ob/B. Pm) (Fig. 2K–O), suggesting that GMFB KO contributes to preventing the impaired bone formation induced by oestrogen deficiency in rats. We also observed a less active osteoclast state in GMFB deletion rats (Fig. 2P, Q), suggesting that slower bone tissue breakdown occurred in GMFB knockout rats.

### Deletion of GMFB in BMSCs leads to decreased adipocyte differentiation

**Fig. 3:**
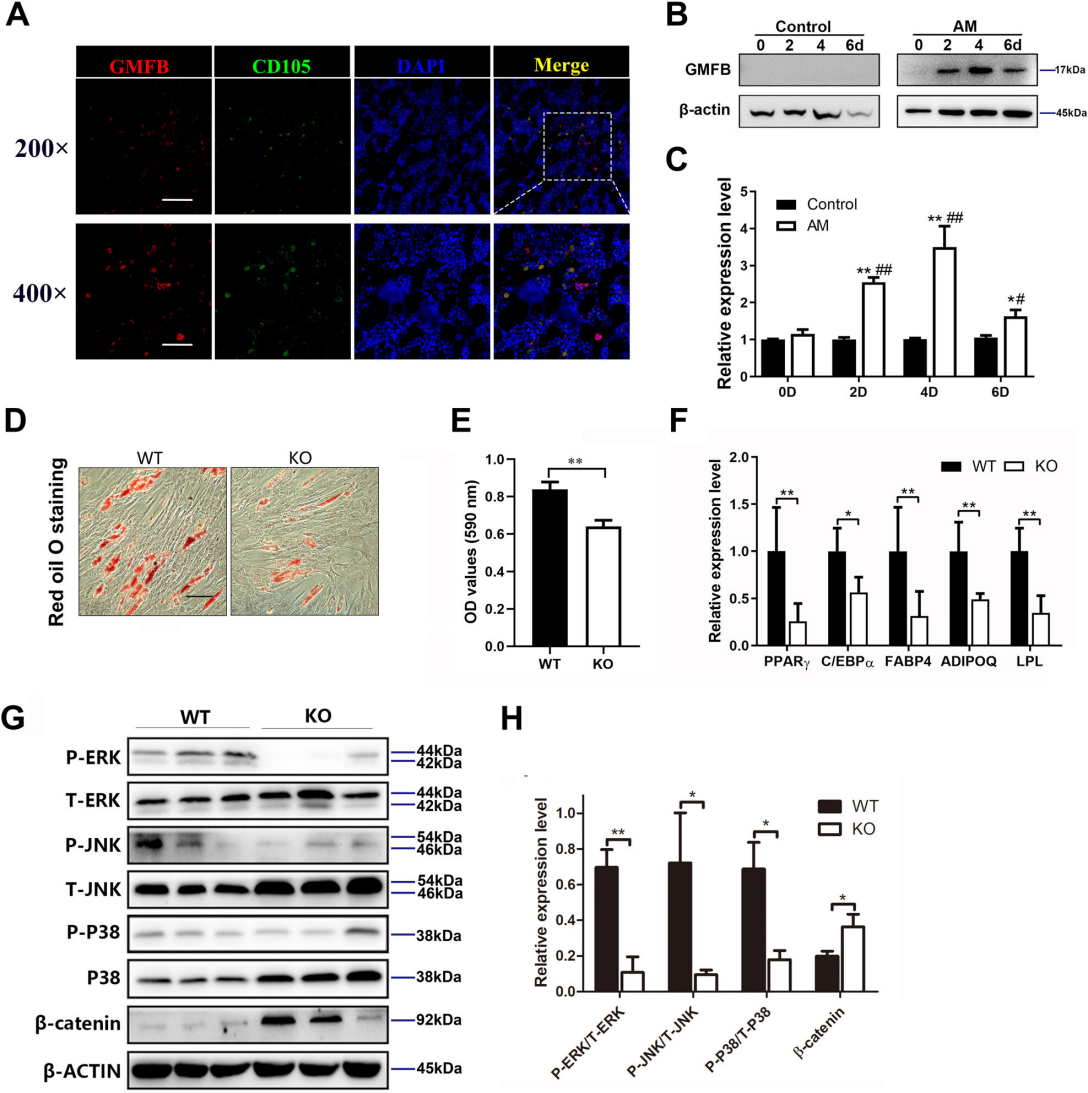
GMFB KO inhibits adipogenesis in BMSCs. **A** Immunostaining of GMFB (red), CD105 (green) and DAPI (blue) in tibia from WT OVX rats (Scale bar, 100 μm (200◊), Scale bar = 50 μm (400◊), n = 5). **B** Western blot analysis of GMFB protein levels in BMSC cultured in growth medium adipogenic medium. **C** Quantification of band density in (**B**). Data represent the mean ± SEM of three independent experiments. Statistical analysis was performed using Student’s t-test. *p < 0.05 compared to respective control group, **p < 0.01 compared to respective control group. #p < 0.05 compared to AM group at 0 weeks, ##p < 0.01 compared to AM group at 0 weeks. **D**, **E** Oil Red O staining (**D**) of BMSCs cultured with adipocyte differentiation medium for 6 days (Scale bar, 50 μm, n = 3). Qquantitative analysis of oil red O staining (**E**). Data are presented as the mean ± SD. **P < 0.01, unpaired Student’s t-test. **F** qPCR analysis of PPARγ, C/EBPα, Fabp4, ADIPOQ and LPL expression in BMSCs from the WT and GMFB KO rats after adipocyte differentiation for 6 days. Data represent the mean ± SD, n = 3. Statistical analysis was performed using unpair Student’s t-test. *p < 0.05 compared to the WT group. **p < 0.01 compared to the WT group. **G** Western blot analysis of MAPK and Wnt signalling pathway protein levels in WT and KO BMSCs after adipocyte differentiation for 6 days. β-actin was used as a reference protein. **H** Quantification of band density in (**E**). Data represents the mean ± SEM of three independent experiments. Statistical analysis was performed using Student’s t-test. *p < 0.05 compared to the WT group. **p < 0.01 compared to the WT group.

We observed that GMFB was highly expressed in the bone marrow (Fig. 3A). CD105 is a marker of BMSCs, and further research revealed that GMFB and CD105 were colocalized (Fig. 3A). These results indicate that GMFB may function in BMSC commitment. The decreased number of adipocytes in the GMFB KO OVX rats prompted us to further explore the role of GMFB in the adipocyte differentiation of BMSCs. We first examined the protein level of GMFB during BMSC adipogenic differentiation. When BMSCs were induced to differentiate into adipocytes, the protein levels of GMFB increased (Fig. 3B, C, Fig. S8). Then, we isolated BMSCs from WT and KO rats to verify their potential for adipogenesis in vitro. BMSCs were cultured in adipogenic medium (AM) for 7 days. Oil Red O staining indicated that adipogenic differentiation was suppressed in the GMFB KO BMSCs compared with the WT BMSCs (Fig. 3D, E).

The expression levels of adipocyte markers (PPARγ, CEBPα, FABP4, ADIPOQ, and LPL) were measured via RT‒PCR. As shown in Fig. 3F, GMFB KO inhibited the expression of adipogenesis-related markers. These data revealed that GMFB deletion inhibited the expression of adipogenic genes and lipid formation in BMSCs.

MAPK and WNT signalling are well-established mechanisms that regulate the adipogenesis of BMSCs, and we investigated whether GMFB KO modulates these pathways. We found that GMFB KO inhibited the activation of the MAPK pathway and promoted the activation of the WNT pathway (Fig. 3G, H, Fig. S9).

### GMFB deletion inhibits PPAR signalling in BMSCs

**Fig. 4:**
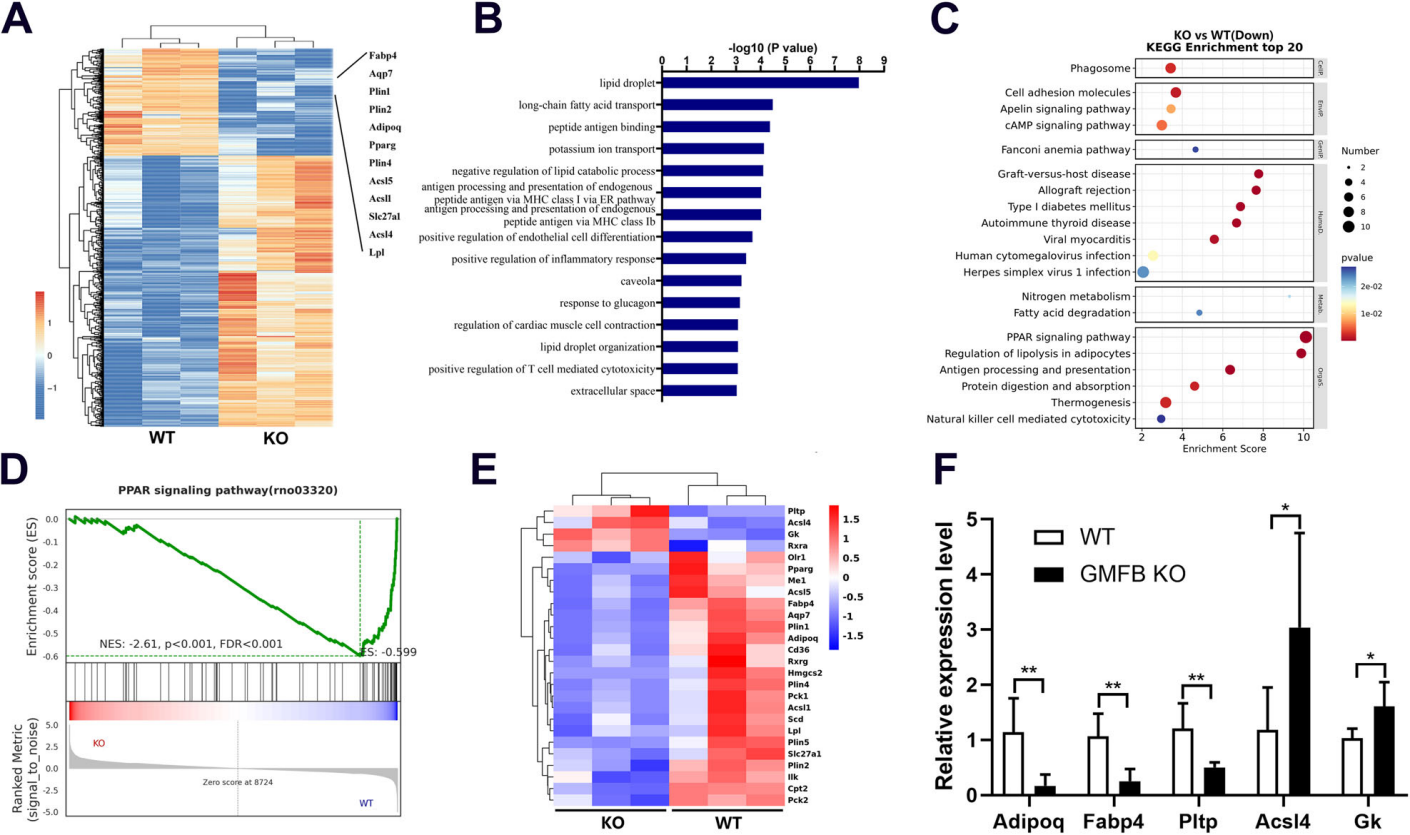
GMFB KO inhibits PPAR signaling in BMSCs. **A** Heatmap of RNA sequencing data between the WT and GMFB KO rat BMSCs cultured in adipocyte differentiation medium for 6 days (n = 3). **B** Downregulated GO analysis associated with significantly regulated genes (P < 0.05) in the GMFB knockout versus WT control groups. **C** Downregulated pathways associated with significantly regulated genes (P < 0.05) in the GMFB knockout versus WT control groups. **D** GSEA of the enrichment of all genes in RNA sequencing. **E** Heatmap showing the gene expression values of indicated genes (RNA-seq) associated with PPAR signalling in comparison with GMFB knockout and WT control groups (n = 3). **F** qPCR results of PPAR signalling-related gene expression in the GMFB KO and WT rat BMSCs. Data represent the mean ± SD, n = 3. *P < 0.05, **P < 0.01, unpaired Student’s t-test.

RNA sequencing analysis was performed to explore the molecular mechanism by which GMFB regulates the adipocyte differentiation of BMSCs. The expression of genes related to adipocyte differentiation was downregulated (Fig. 4A). Gene Ontology (GO) term analysis revealed that lipid droplet, lipid droplet organization and adipocyte differentiation markers were downregulated in the GMFB KO BMSCs (Fig. 4B). Kyoto Encyclopedia of Genes and Genomes (KEGG) pathway analysis was performed to identify significant signalling pathways. Fatty acid degradation, the PPAR signalling pathway and the regulation of lipolysis in adipocytes were significantly inhibited in the GMFB KO BMSCs (Fig. 4C). Furthermore, gene set enrichment analysis (GSEA) revealed that GMFB KO decreased the enrichment score of the PPAR signalling pathway (Fig. 4D). We then compared the PPAR signalling pathway-related genes in adipogenesis BMSCs, and the KO and WT BMSCs presented significantly different patterns of gene expression after adipogenesis was induced (Fig. 4E). The differentially expressed gene (DEG) expression levels were further confirmed by RT-PCR in KO and WT BMSCs after adipogenesis induction (Fig. 4F). As PPARγ is considered one of the major drivers of adipogenesis [20, 24], these results suggest that GMFB KO may inhibit the PPAR signalling pathway to suppress adipogenesis in BMSCs.

### GMFB deletion modulates the remodelling of the actin cytoskeleton in BMSCs

**Fig. 5:**
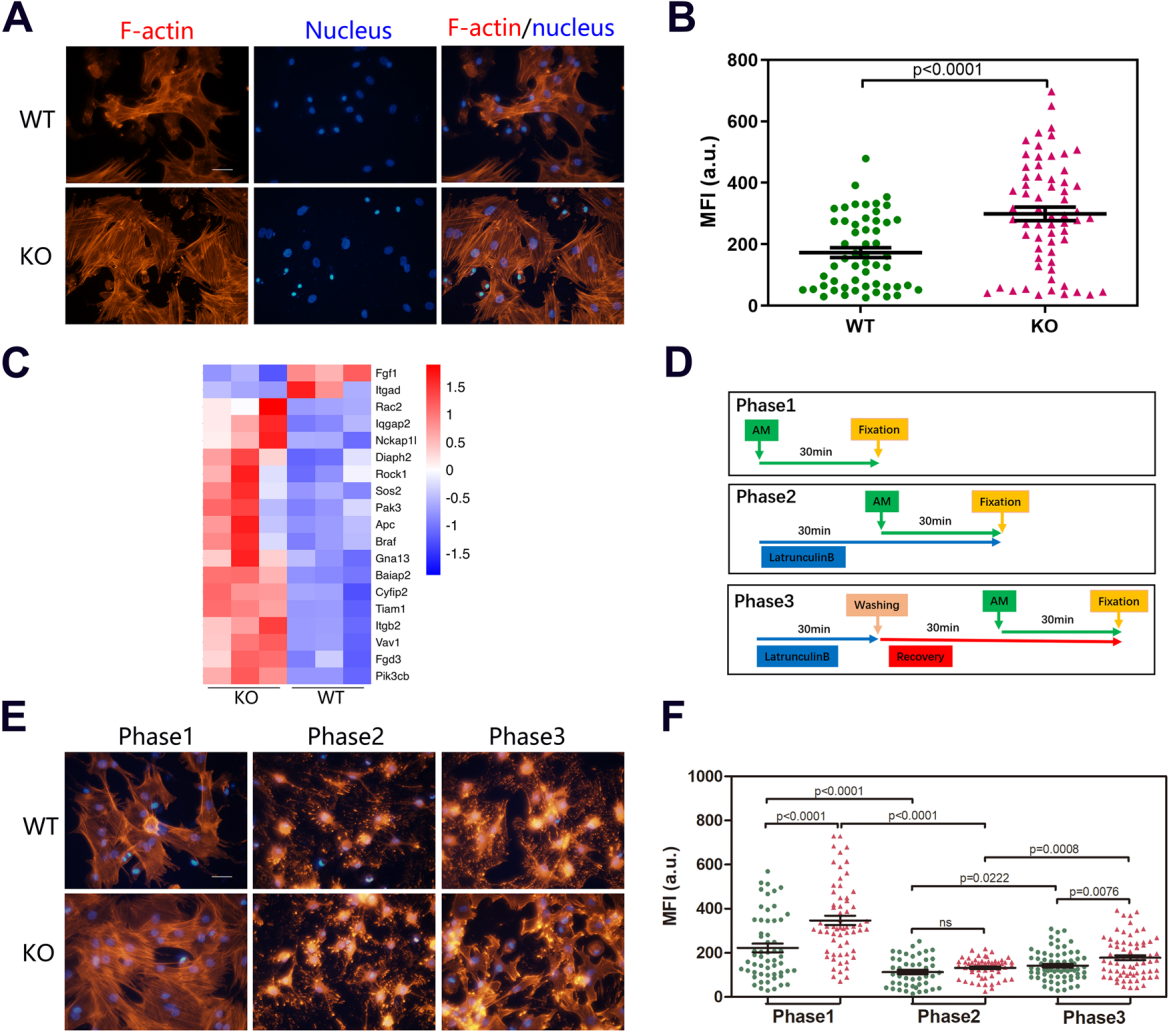
GMFB modulates actin-filament organization in BMSCs. **A**, **B** Representative immunofluorescent images (**A**) in 48 h after seeding BMSCs with F-actin stained (phalloidin, red). Scale bars: 50 μm. Quantification of actin means fluorescent intensity (MFI) (**B**) of 48 h after seeding BMSCs. **C** Heatmap showing the gene expression values of indicated genes (RNA-seq) associated with actin-filament organization in comparison with GMFB KO and WT groups (n = 3). **D**–**F** Schematic for treatment 48 h after seeding of BMSCs with adipocyte differentiation medium and latrunculin B for actin-filament polymerization assay (**D**). Representative immunofluorescent images (**E**) in the cells with F-actin stained (phalloidin, red). Scale bar: 50 μm. Quantification of actin MFI (**F**) of BMSCs following treatment with different phases.

We performed LC‒MS/MS analysis to identify the interaction between GMFB and PPARγ. Unfortunately, we did not detect any physically direct interaction between GMFB and PPARγ (Fig. S10A, B). The data instead revealed an interaction between GMFB and ACTB (Fig. S10C, D). GMFB binds the Arp2/3 complex, promotes the debranching of actin filament networks, and is essential for normal cytoskeleton organization [12]. Therefore, we first observed changes in the actin cytoskeleton resulting from GMFB modulation by staining with phalloidin, which binds to F-actin. At 48 h after seeding, the actin filaments in the WT BMSCs appeared small, short and disordered, whereas in the KO BMSCs, the actin filaments appeared long, straight, and ordered (Fig. 5A). Compared with WT controls, GMFB KO significantly increased the F-actin mean intensity (Fig. 5B). We then compared the DEGs in adipogenic BMSCs and found that KO and WT BMSCs presented markedly different patterns of cytoskeleton-related gene expression after adipogenesis was induced (Fig. 5C).

To determine whether actin‒filament organization differs between KO and WT BMSCs after adipogenesis induction, we treated BMSCs with latrunculin B (2 μg/mL) to disrupt the actin networks and cultured the cells with adipogenesis medium (AM). The process is illustrated in Fig. 5D. We detected the actin cytoskeleton morphology at 3 phases (Fig. 5D): phase 1, baseline of actin-filament organization under AM conditions without latrunculin B treatment; phase 2, depolymerization of actin filaments with latrunculin B treatment; and phase 3, repolymerization of actin filaments with latrunculin B washed off [18]. Imaging analysis revealed that the morphology of the actin cytoskeleton in KO and WT BMSCs differed at phase 1 (Fig. 5E), as we observed (Fig. 5A). The mean F-actin intensity in adipogenesis-induced KO BMSCs was significantly greater than that in WT control BMSCs (Fig. 5F). In phase 2, both KO and WT BMSCs presented a significant absence of actin filaments resulting from latrunculin B treatment (Fig. 5E), and there was no difference between the two groups. In phase 3, 1 h after the latrunculin B was washed off, the KO BMSCs presented markedly greater numbers of actin filaments than the WT BMSCs did. In addition, F-actin mean intensity analysis indicated that the recovery of actin filaments in KO BMSCs was greater than that in WT BMSCs (Fig. 5E).

### The actin cytoskeleton regulates intracellular Ca^2+^ concentration and adipogenesis activation

**Fig. 6:**
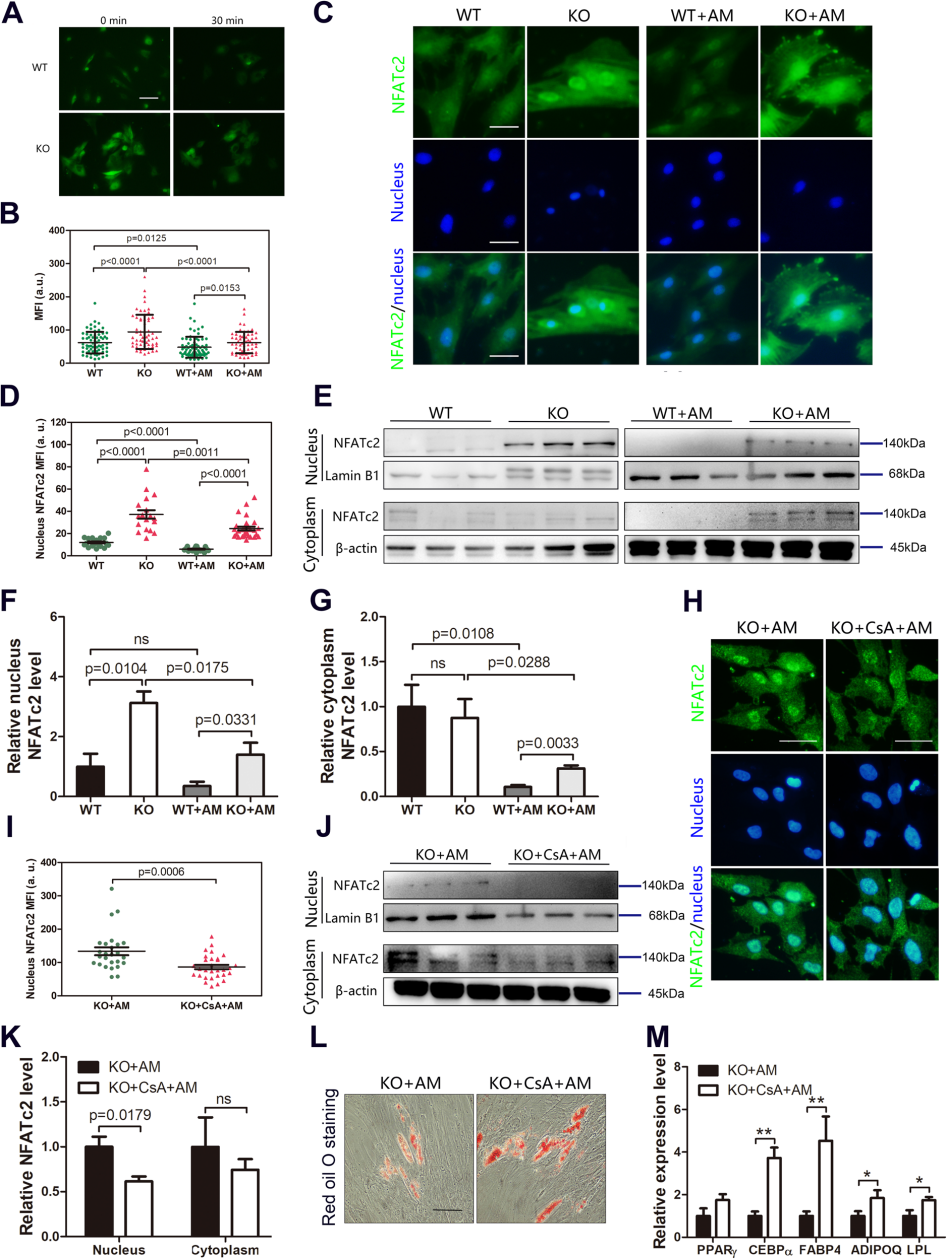
Cytoskeletal regulates Ca^2+^-calcineurin-NFATc2 and adipogenesis activation. **A**, **B** Representative immunofluorescent images of Ca^2+^ (**A**) in BMSCs treated with growth medium or adipocyte differentiation medium for 30 min. Scale bars: 50 μm. Quantification of Ca^2+^ mean fluorescent intensity (MFI) (**B**) of BMSCs treated with growth medium or adipocyte differentiation medium for 30 min. **C** Immunofluorescent staining of NFATc2 (green) in BMSCs treated with growth medium or adipocyte differentiation medium for 48 h. 4′,6- diamidino- 2- phenylindole (DAPI) was used for nucleus staining (blue). **D** Quantification of nucleus NFATc2 mean fluorescent intensity (MFI) of WT and KO BMSCs treated with growth medium or adipocyte differentiation medium for 48 h. **E** Representative western blot for NFATc2 in the nucleus and cytoplasm of WT and KO BMSCs treated with growth medium or adipocyte differentiation medium for 48 h. **F**, **G** Quantification of nucleus (**F**) and cytoplasm (**G**) band density in (**E**). Data represent the mean ± SEM of three independent experiments. Statistical analysis was performed using Student’s t-test. **H** Immunofluorescent staining of NFATc2 (green) in GMFB KO BMSCs treated with adipocyte differentiation medium with or without cyclosporin A for 48 h. 4′,6- diamidino- 2- phenylindole (DAPI) was used for nucleus staining (blue). **I** Quantification of nucleus NFATc2 mean fluorescent intensity (MFI) of GMFB KO BMSCs treated with adipocyte differentiation medium with or without cyclosporin A for 48 h. **J** Western blot for NFATc2 in the nucleus and cytoplasm of GMFB KO BMSCs treated with adipocyte differentiation medium with or without cyclosporin A for 48 h. **K** Quantification of band density in (**J**). Data represent the mean ± SEM of three independent experiments. Statistical analysis was performed using Student’s t-test. **L** Oil Red O staining (**I**) of GMFB KO BMSCs cultured in adipocyte differentiation medium with or without cyclosporin A for 6 days (Scale bar, 50 μm, n = 3). **M** qPCR analysis of PPARγ, CEBPα, FABP4, ADIPOQ, and LPL expression in GMFB KO BMSCs cultured in adipocyte differentiation medium with or without cyclosporin A for 6 days. Data represent the mean ± SD, n = 3. *p < 0.05, **p < 0.01, unpaired Student’s t-test.

We next investigated the role of the actin cytoskeleton in regulating PPARγ activation. A previous study showed that the actin cytoskeleton regulated Ca^2+^ influx [25]. We exposed WT or KO BMSCs to AM and quantified changes in the Ca^2+^ concentration in the cytoplasm. We observed a reduction in the Ca^2+^ concentration in WT BMSCs compared with that in KO BMSCs after adipogenic induction (Fig. 6A, B). These data suggest that stimulation of actin polymerization in GMFB KO mice increased the Ca^2+^ concentration.

Ca^2+^ is the primary regulator of NFATc2 activation, and the Ca^2+^-calcineurin-dependent pathway of NFATc2 nuclear translocation is triggered by an increase in cytoplasmic Ca^2+^, which regulates multiple pathophysiological processes [26]. We observed the NFATc2 distribution in BMSCs after adipogenic induction. Immunofluorescence assays revealed positive staining for NFATc2 in the nucleus of BMSCs, which was translocated to the cytoplasm upon adipogenic induction (Fig. 6C). The nuclear NFATc2 level in the KO BMSCs was greater than that in the WT BMSCs, with or without adipogenic induction (Fig. 6D). The protein level of NFATc2 was also confirmed by Western blot analysis (Fig. 6E, Fig. S11). Consistent with these results, we detected significantly greater protein levels of NFATc2 in the nuclei of the KO BMSCs than in those of the WT BMSCs (Fig. 6E, F). Adipogenesis reduced the nuclear protein level of NFATc2 in both WT and KO BMSCs; however, the nuclear protein level of NFATc2 in the KO + AM BMSCs was still significantly greater than that in the WT + AM BMSCs (Fig. 6F). In addition, there was no difference in the cytoplasmic protein level of NFATc2 between WT and KO BMSCs. Adipogenesis also reduced the cytoplasmic protein level of NFATc2 in both WT and KO BMSCs, and the cytoplasmic protein level of NFATc2 in the KO + AM BMSCs was significantly greater than that in the WT + AM BMSCs (Fig. 6G). A previous study confirmed that NFATc2 directly binds to PPARγ in the nucleus and negatively regulates its transcriptional activity [27].

Next, we used cyclosporin A (CsA), a specific inhibitor of calcineurin, to further confirm the role of GMFB in the calcineurin-NFATc2 pathway. Immunofluorescence analysis revealed that cyclosporin A decreased nuclear NFATc1 in KO BMSCs after adipogenic induction (Fig. 6H, I). The protein level of NFATc2 was also confirmed by Western blot analyses. Cyclosporin A decreased the nuclear protein level of NFATc2 in the KO BMSCs after adipogenesis but had no effect on the cytoplasmic protein level of NFATc2 (Fig. 6J, K, Fig. S12). Oil Red O staining indicated that adipogenic differentiation was stimulated in the cyclosporin A-treated KO BMSCs compared with the KO BMSCs (Fig. 6L). Compared with those in KO BMSCs, the expression levels of adipocyte markers such as PPARγ, C/EBPα, FABP4, ADIPOQ, and LPL were increased in cyclosporin A-treated KO BMSCs upon induction of adipogenesis (Fig. 6M). Therefore, GMFB deletion inhibited the adipogenesis of BMSCs via the Ca^2+^-calcineurin-NFATc2 pathway.

### GMFB downregulation partially rescues the bone loss induced by OVX

**Fig. 7:**
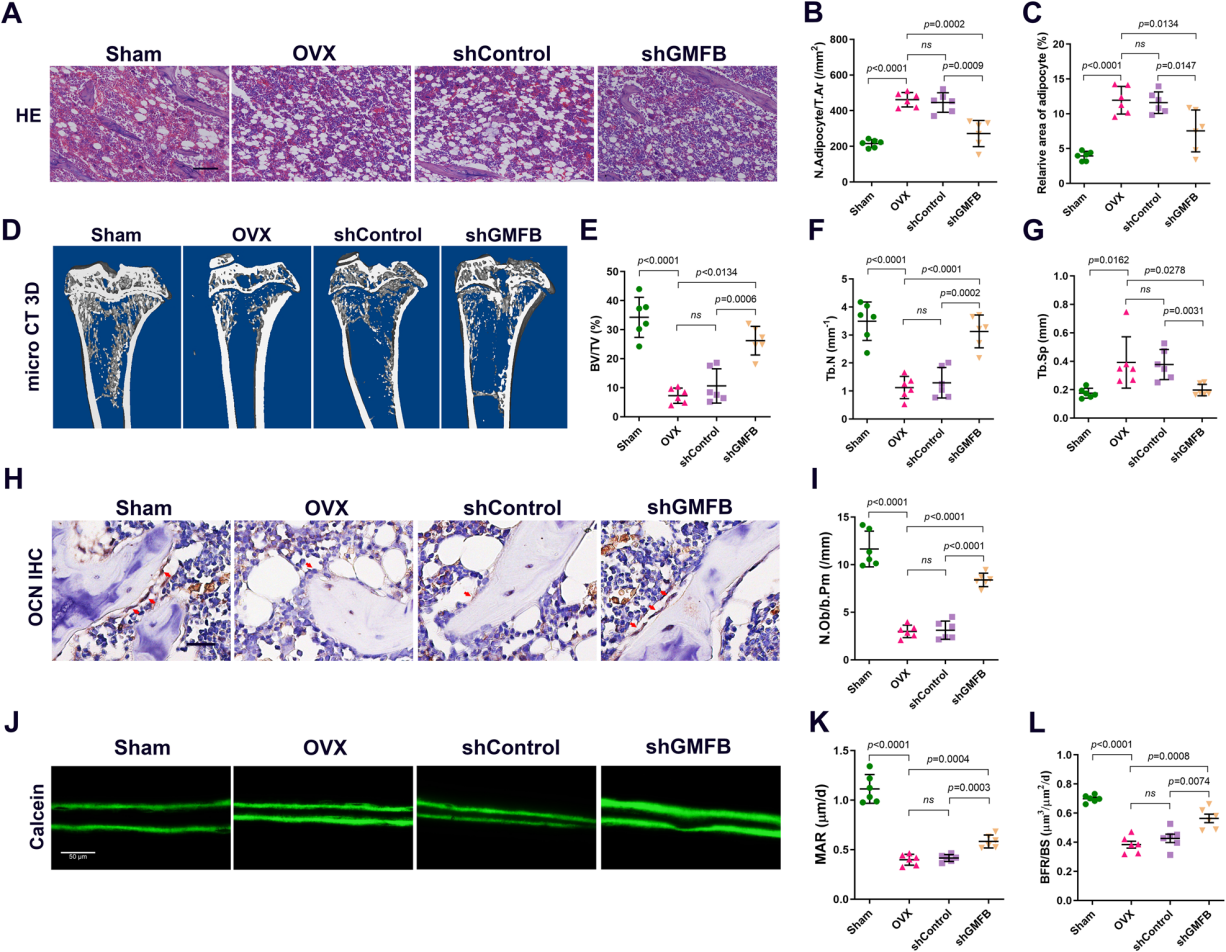
Downexpression of GMFB partially rescued the bone loss induced by OVX. **A**–**C** Representative images of H&E staining (**A**) and quantification of adipocyte number (**B**) and area (**C**) in rat tibias from Sham, OVX, shControl and shGMFB groups. (scale bars, 100 μm, n = 6). **D**–**G** Representative micro-CT images (**D**) and quantification of trabecular bone volume per tissue volume (Tb.BV/TV) (**E**), trabecular number (Tb.N) (**F**) and trabecular separation (Tb.Sp) (**G**) in tibias (n = 6). **H** and **I** Representative images of osteocalcin (OCN) staining (**H**) and quantification of number of OCN^+^ cells (red arrows) in tibias (**I**) (scale bars, 50 μm, n = 6). **J**–**L** Representative images of calcein double-labelling (**J**) and analysis of mineral apposition rates (MARs) (**K**) and Bone formation rate per unit of bone surface (BFR/BS) (**L**) of trabecular bone in tibias (scale bars, 50 μm, n = 6).

Since the deletion of GMFB in rats prevents oestrogen deficiency-induced osteoporosis, we next investigated whether the knockdown of GMFB expression would rescue the bone phenotype of OVX rats. We utilized rAAV9 gene delivery of GMFB shRNA (rAAV9-shRNA-Cherry) to suppress the expression of GMFB in OVX rats via intraosseous injection. The shRNA and Gmfb overexpression plasmids were used to cotransfect 293T cells in vitro, and fluorescence attenuation tests indicated that the shRNA effectively inhibited GMFB expression (Fig. S13A). The knockdown of GMFB by injection of shRNA virus in OVX rat bone was validated by Western blotting (Fig. S13B). We found that GMFB knockdown in OVX rats partially alleviated OVX-induced bone fat accumulation (Fig. 7A–C). Compared with rAAV9-Cherry control rats, OVX rats injected with rAAV9-shRNA-Cherry presented increased bone mass (Fig. 7D–G) and osteoblast numbers (Fig. 7H, I). In addition, OVX rats treated with shGMFB had faster MAR and BFR than control rats did (Fig. 7J–L).

## Discussion

The pathological bone loss conditions associated with oestrogen deficiency are characterized by increased bone marrow adipose tissue (BMAT) accumulation and compromised bone formation [28, 29]. This could be largely attributed to the shift in the balance of BMSC differentiation towards adipogenesis under oestrogen deficiency. Previous studies have revealed an increased capacity to differentiate into adipocytes and a reduced capacity to differentiate into osteoblasts in BMSCs isolated from postmenopausal osteoporotic subjects [30–32]. However, the molecular mechanisms underlying this cellular event are not fully understood.

GMFB is conserved from yeast to mammals and is expressed in diverse organs and tissues[12, 33]. We found that the expression of GMFB was significantly greater in bone samples from osteoporotic patients than in those from healthy controls (Fig. 1D, E). The protein levels of GMFB in patients and T scores were negatively correlated. Moreover, GMFB expression increased significantly in rat bone tissue after OVX. These data suggest that the expression of GMFB could be necessary for PMOP and that GMFB has a significant promotional role in the process of PMOP.

GMFB is primarily isolated from astrocytes, and numerous studies have focused on the role of GMFB in neurological disorders [34]. However, whether GMFB participates in osteoporosis is unknown. We detected no physiological expression of GMFB in rat bone tissue, which suggests that GMFB may not be an essential element for physiology and may only play a role in pathology. Moreover, there is no data on GMFB expression in various cells in bone tissue. Therefore, in the present study, we used an unconditional GMFB knockout rat model to investigate the function of GMFB in osteoporosis. Here, we provide the first evidence that GMFB KO has a significant positive effect on rescuing OVX-induced increases in bone marrow adipose tissue and bone loss. GMFB KO plays a significant protective role in the process of bone loss induced by OVX.

BMSCs are the common ancestor lineage of bone marrow adipocytes and osteoblasts and a balance exists between these two types of cells. The biased commitment of BMSCs to the osteoblast lineage directly compromises adipogenesis, and vice versa [35]. Oestrogen deficiency results in increased adipogenesis at menopause, which leads to increased BMAT and reduced bone mass [36]. We determined that GMFB was pathologically localized in BMSCs from OVX rats. Therefore, an analysis of the status of adipogenesis in GMFB KO BMSCs was prioritized.

Here, we found that GMFB knockout suppressed the adipogenesis of BMSCs. GMFB knockout activated the Wnt/β-catenin signalling pathway and suppressed the MAPK signalling pathway, which is thought to regulate the adipogenesis of BMSCs [37]. Furthermore, RNA sequence data indicated that GMFB knockout suppressed the PPAR signalling pathway, which has been reported to be a pivotal nuclear receptor that drives adipogenesis in BMSCs [38]. The Wnt/β-catenin pathway has been shown to inhibit PPARγ mRNA expression and may interact with PPARγ signalling [39]. Wnt3a downregulates the expression of C/EBPα and PPARγ to inhibit adipogenic differentiation in rMSCs [40]. Increased levels of β-catenin have been shown to suppress the expression of PPARγ at an early stage of stem cell development [41]. The extracellular-signal-regulated kinase (ERK), c-Jun N-terminal kinase (JNK), p38, and MAPK signalling pathways are known to contribute to adipocyte growth and differentiation and activate various transcription factors. In particular, ERK activation, which is involved in cell cycle progression, is known to be essential for the induction of adipogenesis [42]. ERK differentiates preadipocytes into mature adipocytes by increasing the expression of C/EBPα, C/EBPβ and PPARγ [42]. p38 MAPK promotes cell differentiation in the early stage of adipogenesis [43]. BMP2 activates the ERK and p38 signalling pathways and contributes to the transcriptional activation of PPARγ and C/EBPα, resulting in the promotion of adipogenesis [44, 45]. JNK is implicated in the development of obesity-related insulin resistance [46]. Theobromine inhibits adipocyte differentiation during the early stages of adipogenesis by regulating the expression of PPARγ and C/EBPα through the ERK/JNK signalling pathways in 3T3-L1 preadipocytes[47].

Unexpectedly, we did not find any physically direct interaction between GMFB and PPAR signalling, which is concordant with the database information from BioGRID4.4 (https://thebiogrid.org/109026 or 203523), indicating that GMFB and PPAR do not belong to the interactor lists of each other. GMFB is reported to be a regulator of the actin cytoskeleton with a unique role in actin cytoskeleton remodelling [12, 13]. GMFB binds the Arp2/3 complex and catalyses the debranching of actin filament networks [12]. The cytoskeleton is crucial for transducing mechanical stimuli, generating membrane tension, and maintaining cellular homeostasis and development [48]. In addition, the dynamics of the actin cytoskeleton regulate pronounced changes in cell shape, which is a hallmark of differentiation [6]. Previous results have indicated that adipocyte differentiation required a shift in the actin cytoskeleton structure from filamentous actin (F-actin) to monomeric actin (G-actin) [49–51]. Our data show that GMFB KO increased the mean F-actin intensity of BMSCs. Actin depolymerization stimulates the p38 and ERK1/2 pathways and upregulates the gene expression of PPARγ during adipogenesis [52]. Another study showed that the p38 MAPK and ERK1/2 pathways regulate adipogenic and osteogenic differentiation through the remodelling of actin filaments [8]. These results suggest that GMFB may regulate the adipogenesis of BMSCs via actin cytoskeleton remodelling.

**Fig. 8:**
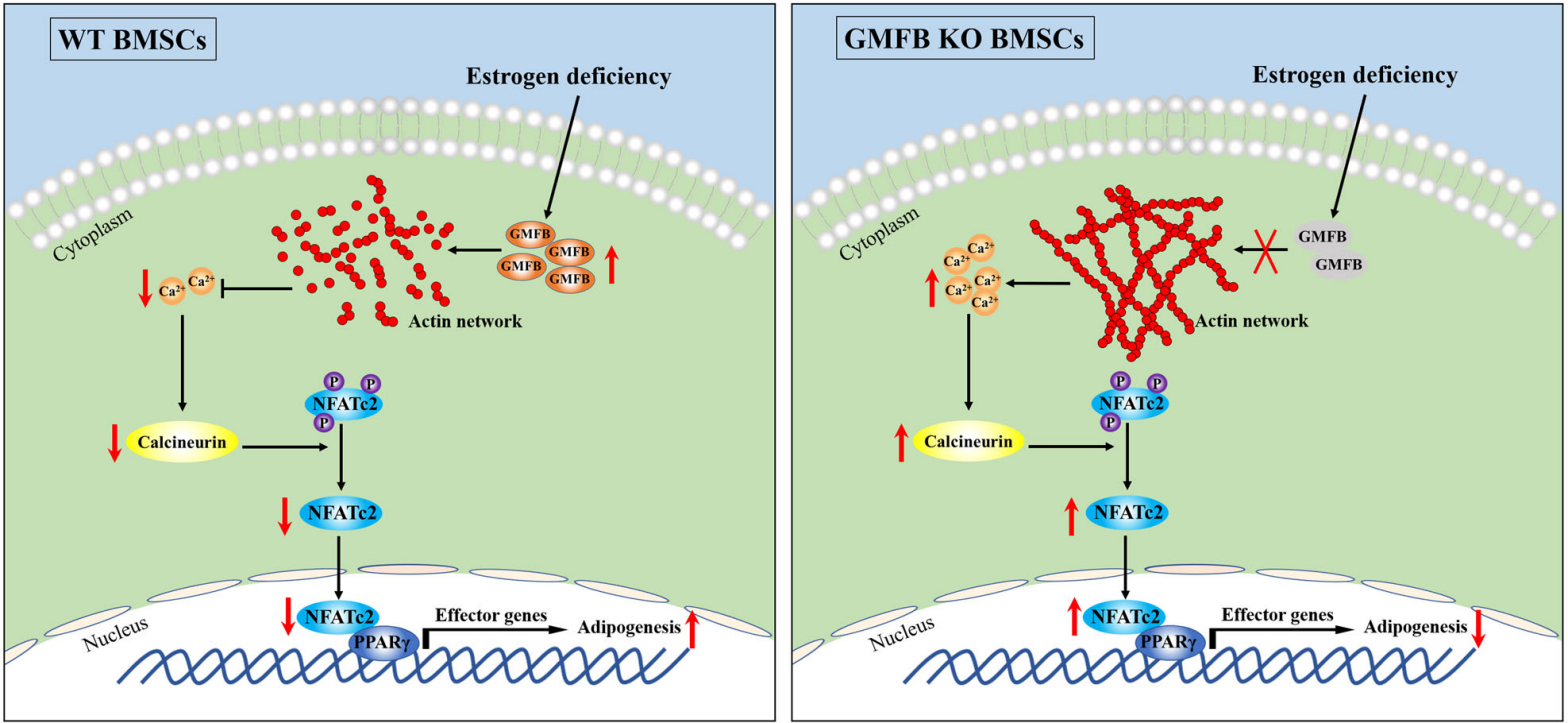
Schematic depicting the function of GMFB in adipogenesis of BMSC in PMOP. In postmenopause female (estrogen deficiency), GMFB stimulates filament debranching and inhibits actin polymerization, which decrease intracellular Ca^2+^ concentration and inhibit Ca^2+^-calcineurin-NFATc2 pathway. Decreased NFATc2 nucleo-cytoplasmic shuttling stimulates NFATc2/PPARγ-dependent adipogenesis-related gene transcription. Gmfb deficiency suppresses adipogenesis of BMSCs via remodelling of the actin cytoskeleton and activation of the Ca^2+^-calcineurin-NFATc2 pathway.

We also showed that GMFB KO enhances F-actin formation and that actin polymerization increases the Ca^2+^ concentration, which is consistent with what has been observed in macrophages [25]. During the early phase of preadipocyte differentiation, increases in the intracellular Ca^2+^ concentration act to potently inhibit adipogenesis [53, 54]. Calcineurin is a calcium-dependent serine/threonine phosphatase and a critical downstream effector of the calcium signal in various cell types [55]. The efficiency of adipogenesis has been shown to be enhanced via the inhibition of endogenous calcineurin activity in 3T3-L1 cells [56]. Ca^2+^ is the primary regulator of NFATc2 activation, and the Ca^2+^-calcineurin-dependent pathway of NFATc2 nuclear translocation is triggered by an increase in cytoplasmic Ca^2+^, which directly binds to PPARγ in the nucleus and negatively regulates its transcriptional activity [27]. The nuclear NFATc2 level in the KO BMSCs was greater than that in the WT BMSCs. Inhibition of calcineurin activity with cyclosporin A suppressed NFATc2 nuclear translocation and blocked the anti-adipogenesis effect of GMFB KO. This molecular evidence can be used to interpret the results of this study and thus could be the molecular mechanism underlying the role of GMFB in the adipogenesis of BMSCs and the occurrence of osteoporosis (Fig. 8).

In light of the high expression of GMFB and the protective effect of GMFB deletion in OVX rats, we investigated the antiosteoporotic effect of GMFB knockdown in vivo in OVX rats by injecting shGMFB or shControl intermittently for >6 weeks. shGMFB markedly protected against bone loss in OVX rats. Additionally, the changes in the BMAT and bone formation indices indicate that the effect of shGMFB may be due to the inhibition of adipogenesis and promotion of bone formation.

There are several limitations to our study. First, as the Gmfb knockout was not cell type-specific in this study, the effects of GMFB KO in other cell types in PMOP are not known. Although GMFB and BMSCs were colocalized in OVX rats, GMFB may play an important role in other cell types in PMOP. Second, we demonstrated that GMFB deletion modulates the remodelling of the actin cytoskeleton and subsequently activates the Ca^2+^-calcineurin-NFATc2 axis in BMSCs; however, the molecular mechanisms by which the actin cytoskeleton regulates the intracellular Ca^2+^ concentration are still poorly understood. Third, the patient sample size was too small to fully represent the actual female population with osteoporosis. A larger sample of osteoporotic patients is needed to confirm our results. Despite these limitations, the significant involvement of GMFB in PMOP warrants further observation to clarify these details.

In conclusion, GMFB expression is significantly increased in bone tissue from osteoporotic patients and OVX rats. Using a systemic knockout approach, we demonstrated that GMFB deficiency significantly improved the phenotype of PMOP by suppressing the adipogenesis of BMSCs. GMFB deficiency positively mediated the activation of the Ca^2+^-calcineurin-NFATc2 axis and subsequently suppressed the function of PPARγ by directly remodelling actin‒filament organization in BMSCs. Local suppression of GMFB rescued the bone loss induced by OVX. GMFB may serve as a potential therapeutic target for treating PMOP.

## Supplementary information

Supplemental materials

## Data Availability

Data are available upon reasonable request. All data and material generated in this study are available upon request from the corresponding author.
