# Supplementary material for: Glial maturation factor-β deficiency prevents oestrogen deficiency-induced bone loss by remodelling the actin network to suppress adipogenesis of bone marrow mesenchymal stem cells
Source: Cell Death Dis. 2024 Nov 14;15(11):829. doi: 10.1038/s41419-024-07234-z (PMC11564563; doi:10.1038/s41419-024-07234-z)
Supplement: Supplementary file 1 — Supplemental materials [file 41419_2024_7234_MOESM1_ESM.docx]

Supplementary Materials for

**Glia maturation factor-β deficiency prevents estrogen deficiency-induced bone loss via remodeling actin network to suppress adipogenesis of bone marrow mesenchymal stem cells**

Jun Xu *et al*

Corresponding author. Email: Huijie Gu, guhuijie0110@126.com; Lixia Lu, lulixia@tongji.edu.cn; Xiaofan Yin, yxf_mh2011@163.com.

**This file includes:**

Figures S1 to S13

Tables S1 to S4


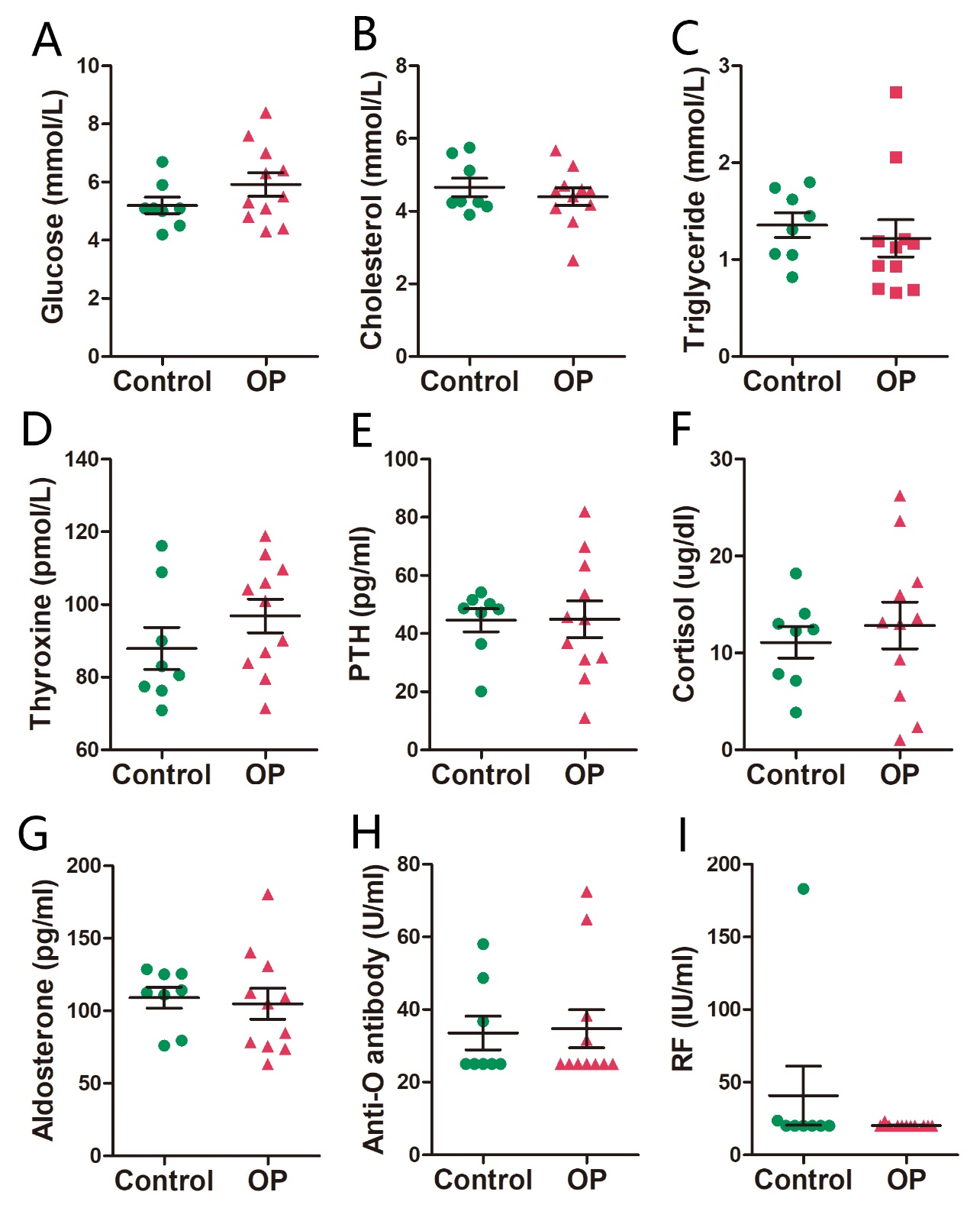


**Figure S1. Serum parameters of glucose metabolism, lipid metabolism endocrine and immune diseases in PMOP patients and controls.**

(A-C) Blood glucose (A), cholesterol (B) and triglyceride (C) in PMOP patients and controls.

(D-F) Thyroxine (D), PTH (E), cortisol (F) and aldosterone (G) in PMOP patients and controls.

(H and I) Anti-O antibody (H) and rheumatoid factor (RF) (I) in PMOP patients and controls.

**
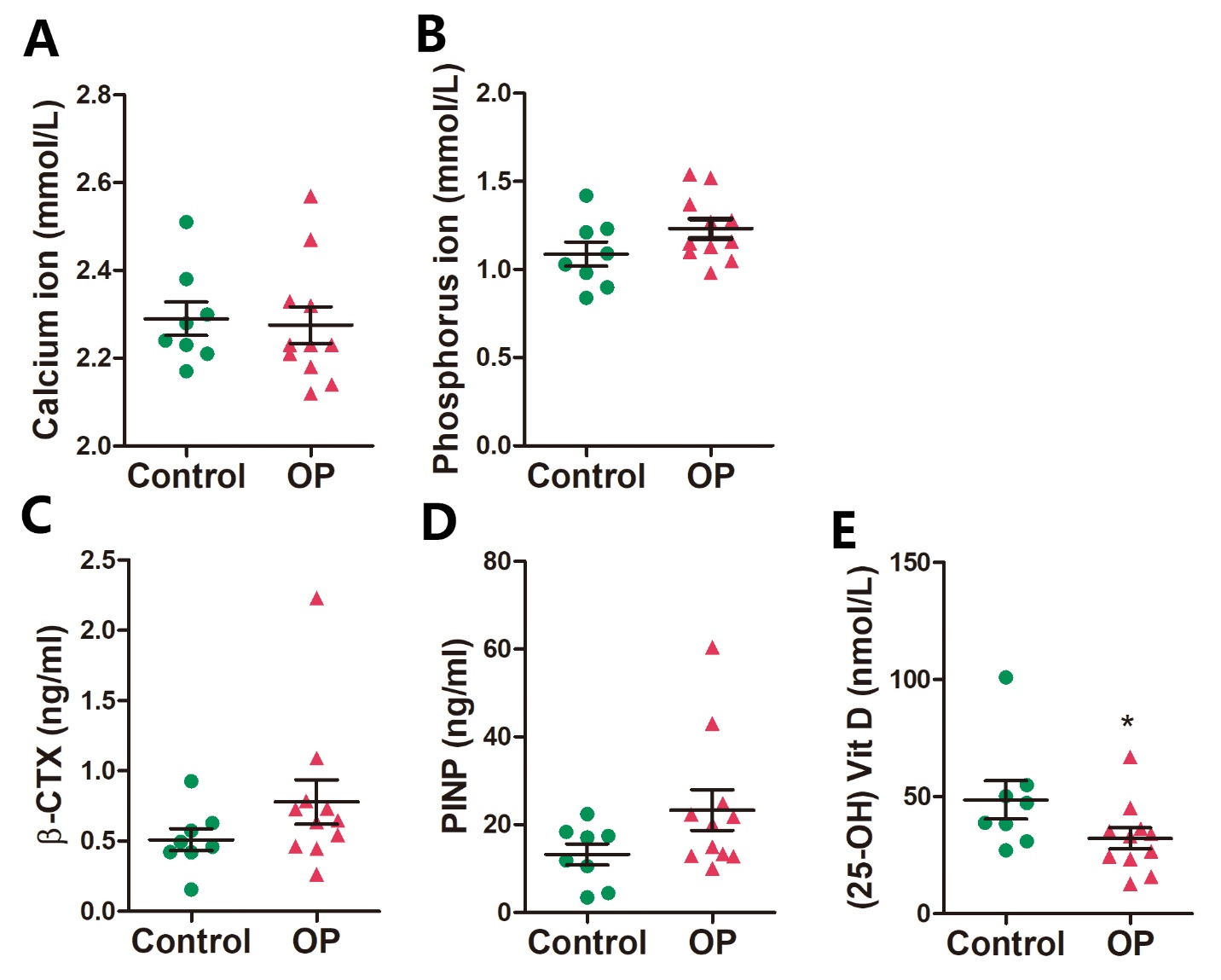
**

**Figure S2. Bone metabolic markers in PMOP patients and controls.** Calcium (A), phosphorus (B), β-CTX (C) and PINP (D), and (25-HO) Vit D (E) in PMOP patients and controls. *P < 0.05, unpaired Student’s t test.

**
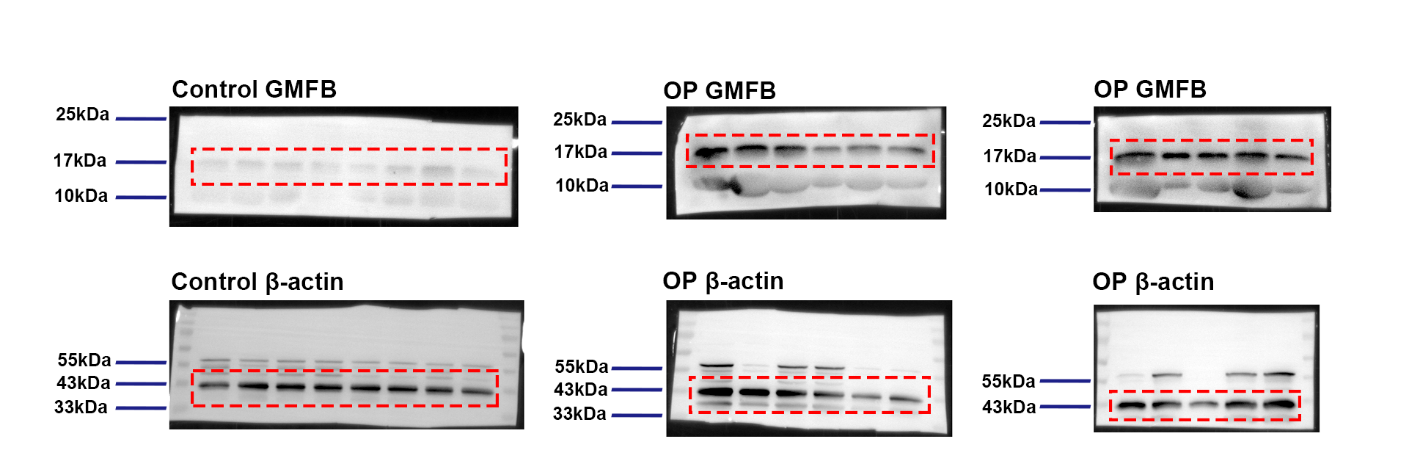
**

**Figure S3. Full and uncropped western blots of Figure 1D.**

**
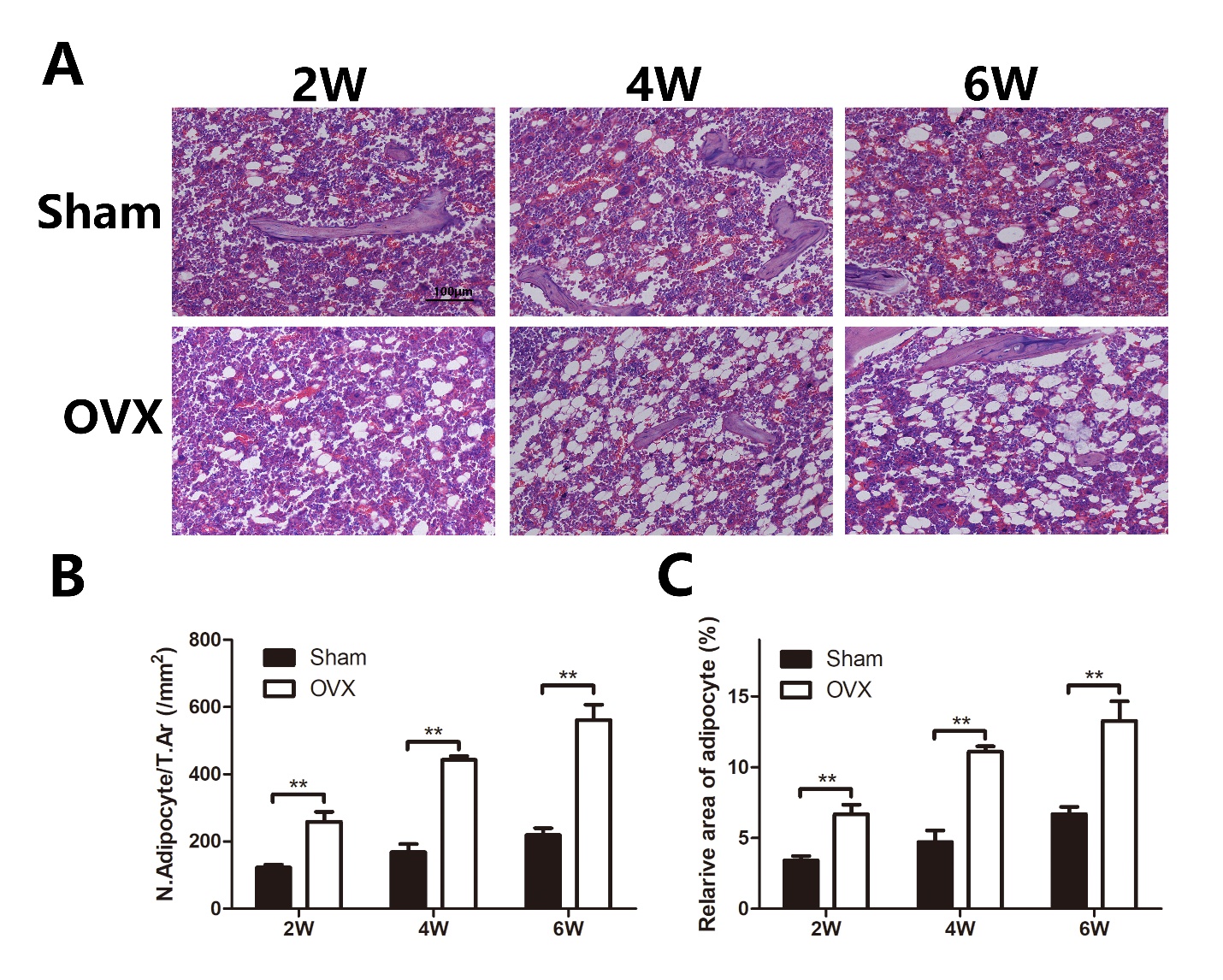
**

**Figure S4. Increased BMAT in OVX induced OP in vivo.**

(A) Representative images of H&E staining in tibias from OVX and Sham female rats.

(B and C) Quantification of adipocyte number (B) and area (C) in tibias from OVX and Sham female rats at 2, 4, and 6 weeks after operation. (scale bars, 100 μm).

**
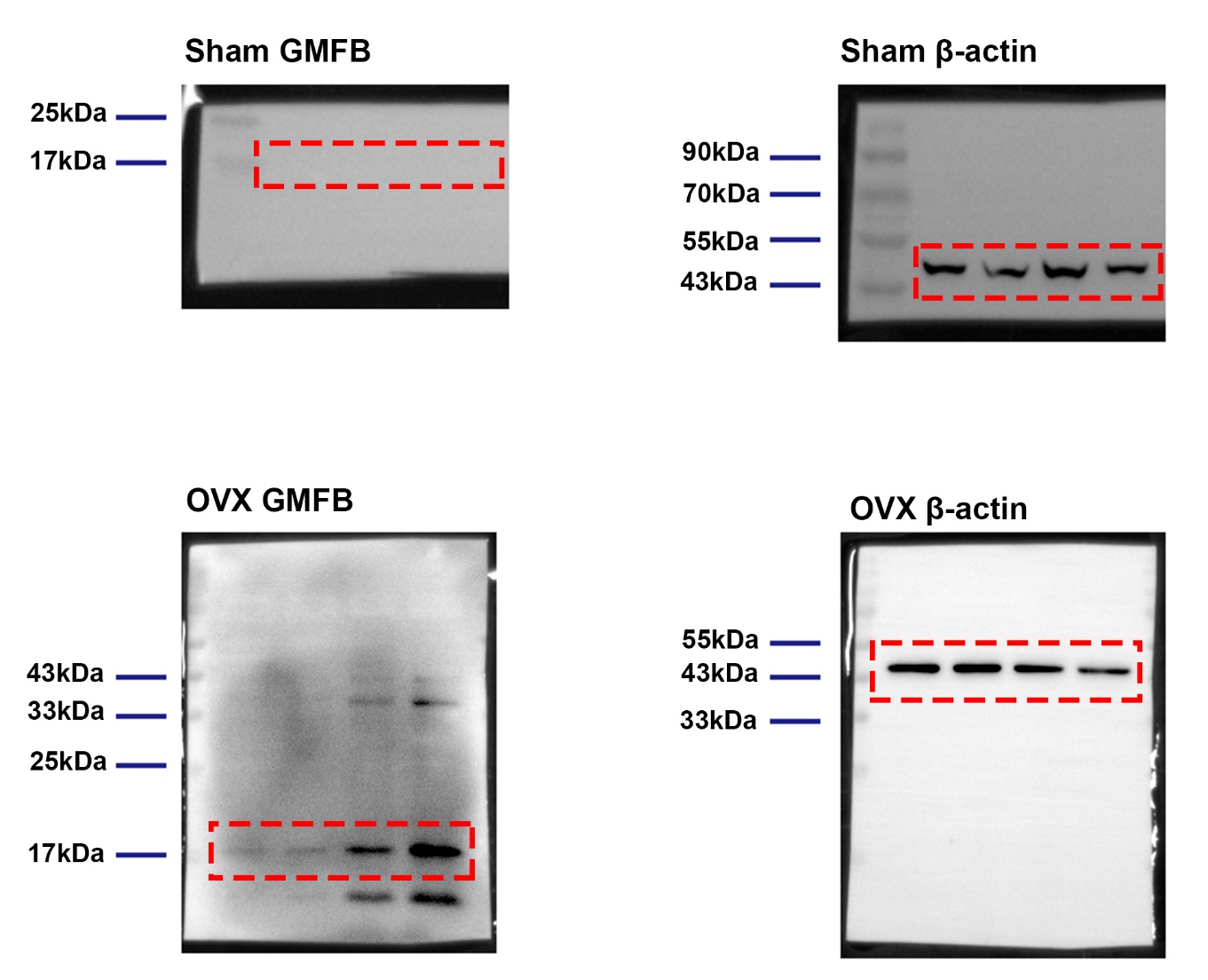
**

**Figure S5. Full and uncropped western blots of Figure 1N.**

**
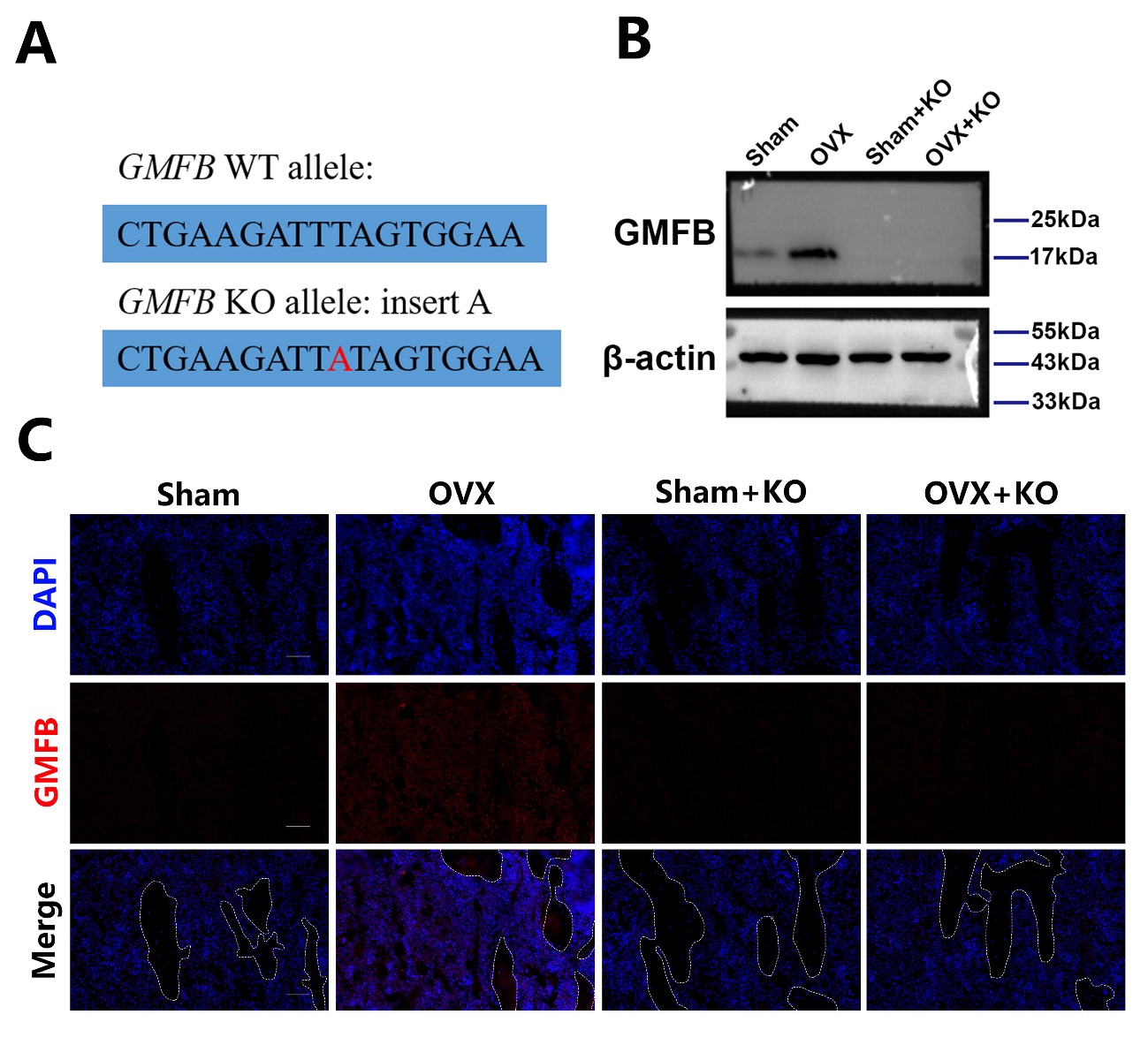
**

**Figure S6. Gmfb deficiency regulated overt phenotypic features in OVX rats.**

(A) One base (a) was inserted into the sequence of gmfb gene on exon 2, resulting in the early termination of GMFB protein translation.

(B) Western blot of GMFB in the bone tissues of tibias from WT and GMFB KO female rats treated with either sham or OVX 4 weeks.

(C) Representative images of GMFB immunofluorescence staining in tibias from WT and GMFB KO female rats treated with either sham or OVX 4 weeks.

**
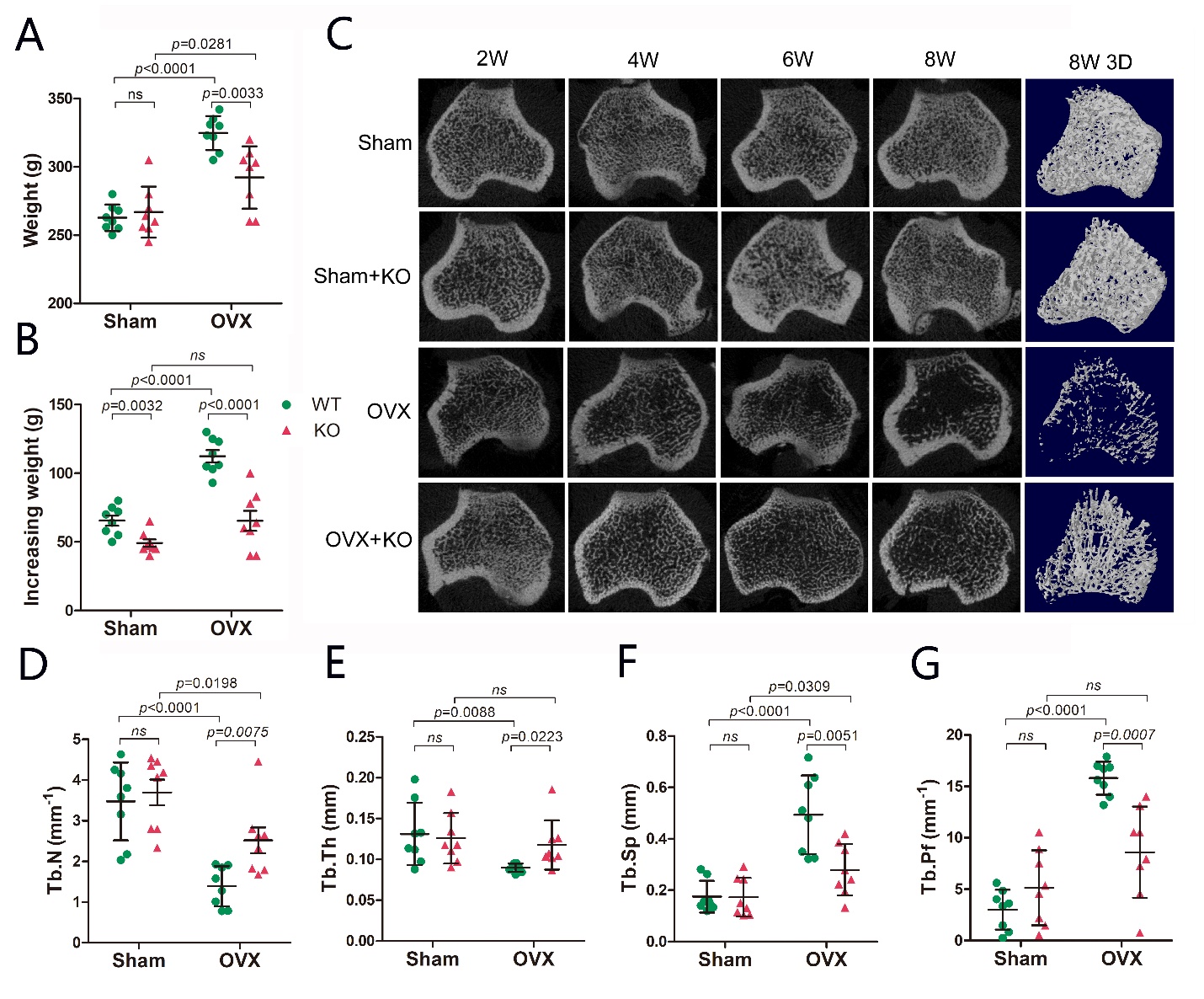
**

**Figure S7. GMFB KO attenuated the osteoporotic phenotype in OVX induced OP in vivo.**

(A and B) Body weight and increased body weight in WT and GMFB KO rats treated with either sham or OVX 8 weeks.

(C) Representative micro-CT images in tibias from WT and GMFB KO female rats treated with either sham or OVX 2, 4, 6 and 8 weeks.

(D-G) Quantification of trabecular number (Tb.N) (D), trabecular thickness (Tb.Th) (E), trabecular separation (Tb.Sp) (F), and trabecular bone pattern factor (Tb.Pf) (G) in tibias from WT and GMFB KO female rats treated with either sham or OVX 8 weeks.


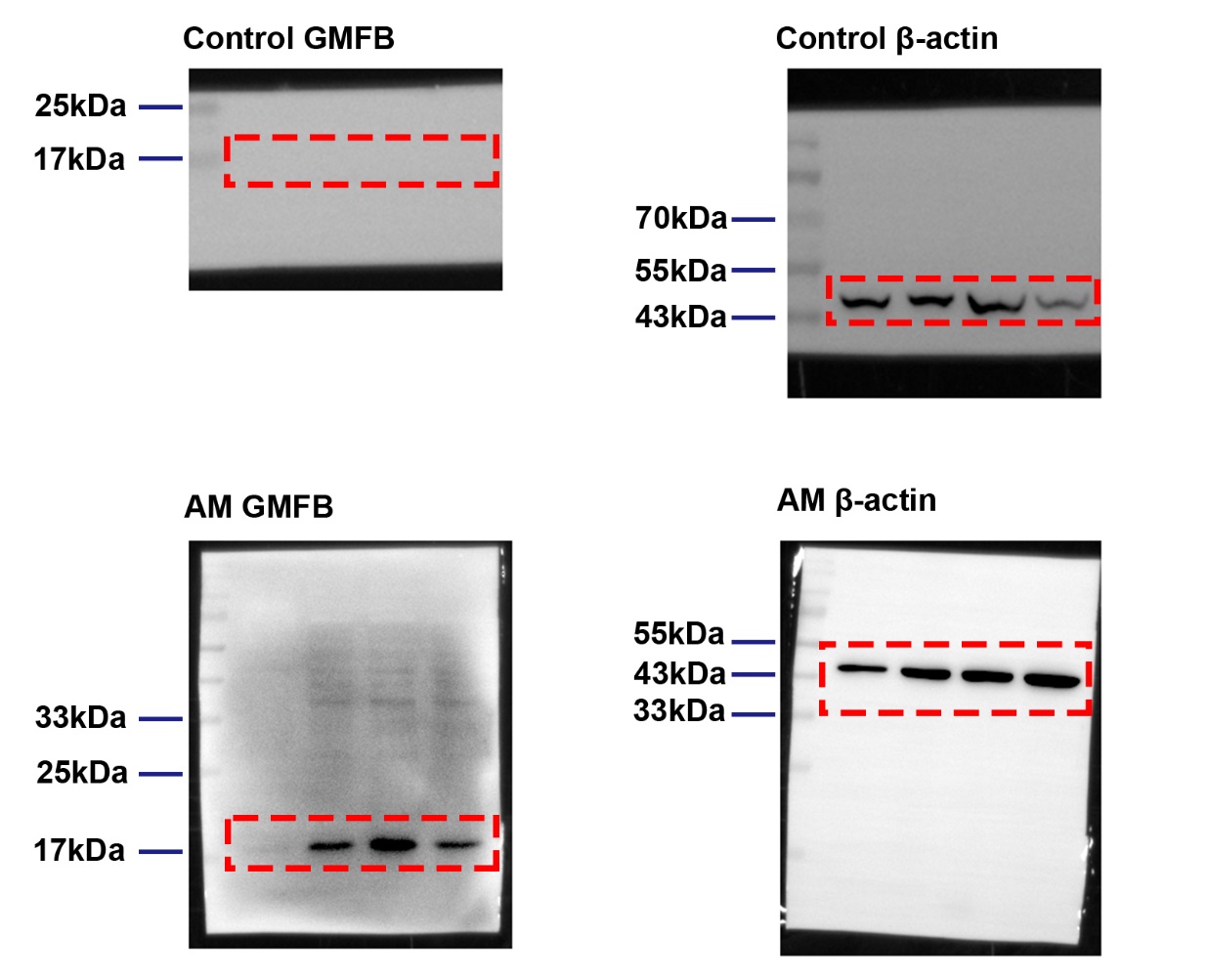


**Figure S8. Full and uncropped western blots of Figure of Figure 3B.**


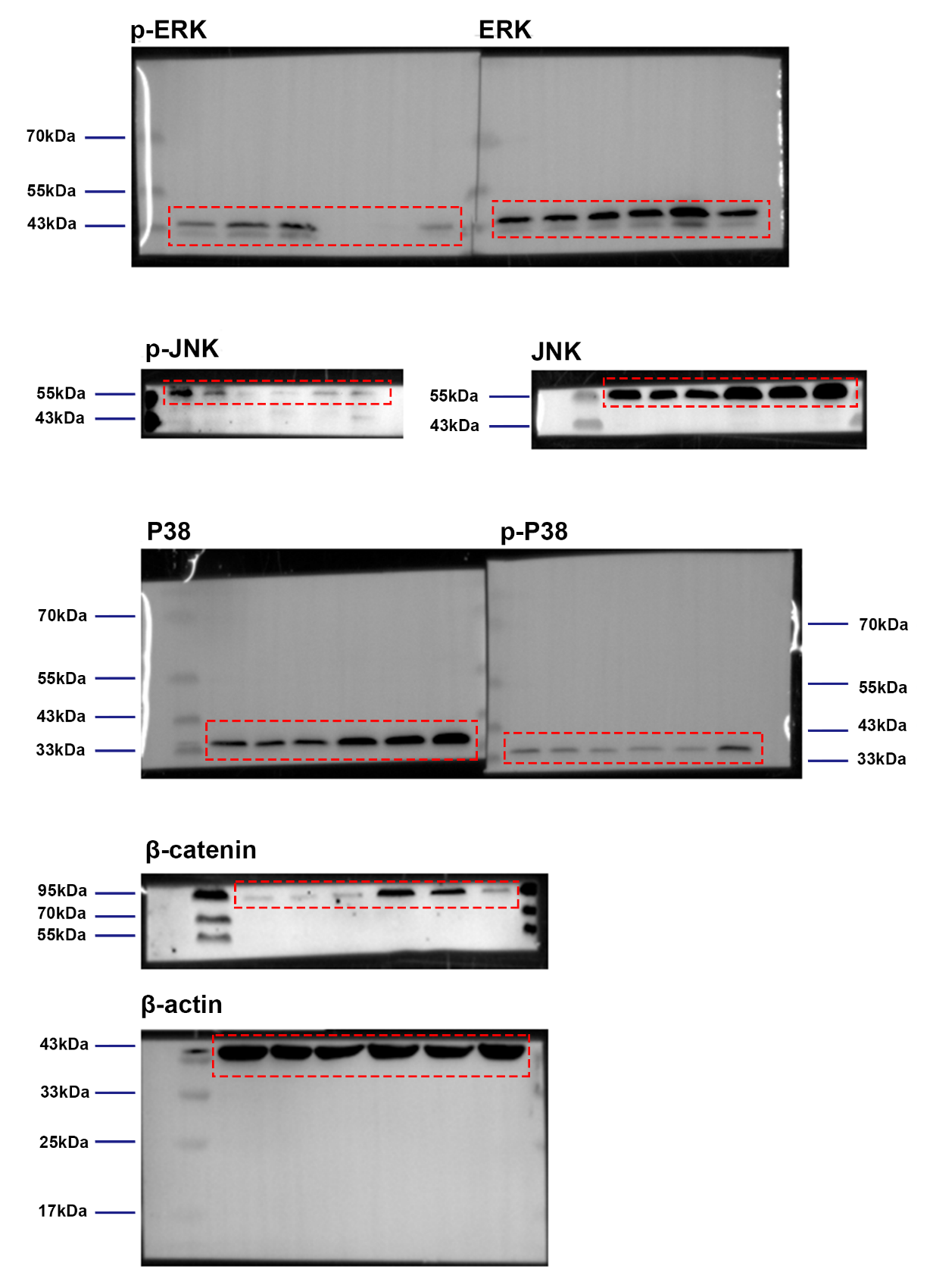


**Figure S9. Full and uncropped western blots of Figure 3G.**


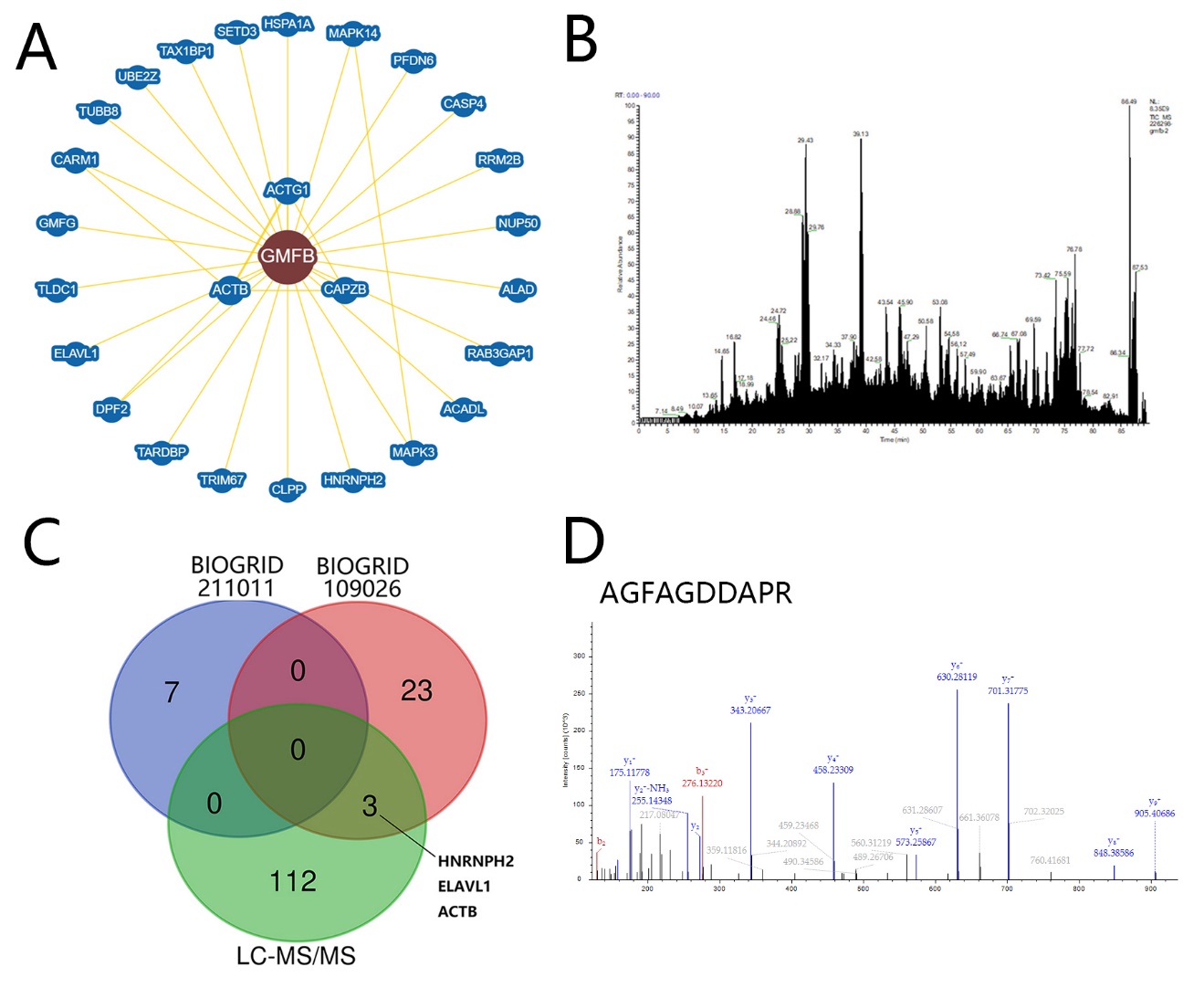


**Figure S10. Interaction between GMFB and ACTB.**

(A) GMFB interacting protein network diagram in the BioGRID4.4 Q24 database (https://thebiogrid.org/109026).

(B) LC-MS/MS identified the total ion flow diagram of GMFB interacting proteins.

(C) Venn diagram of GMFB interacting proteins in our data and the database information from BioGRID4.4 (https://thebiogrid.org/ 109026 or 203523)

(D) Mass spectrogram of identified GMFB binding to ACTB.


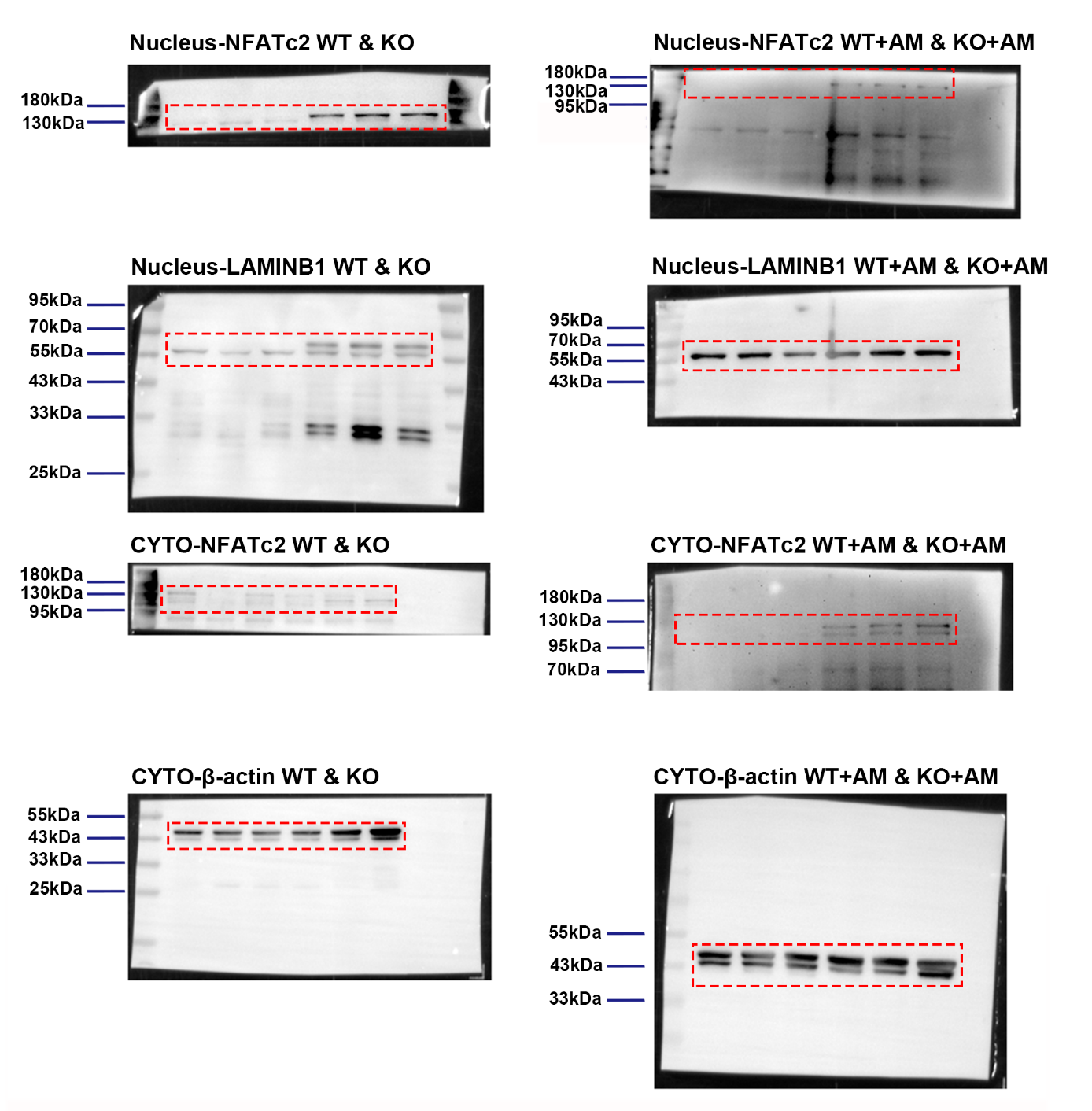


**Figure S11. Full and uncropped western blots of Figure 6E.**


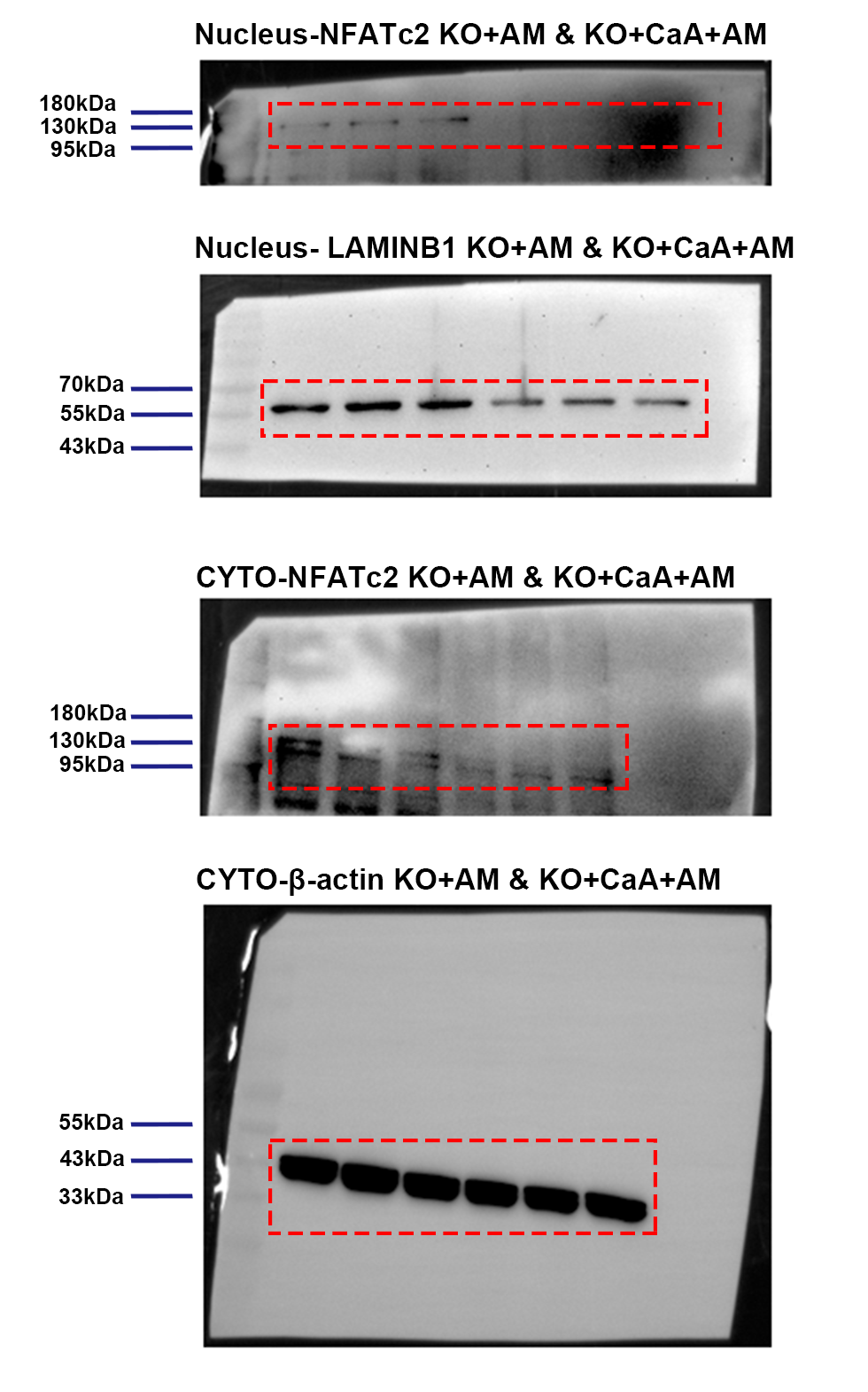


**Figure S12. Full and uncropped western blots of Figure 6J.**


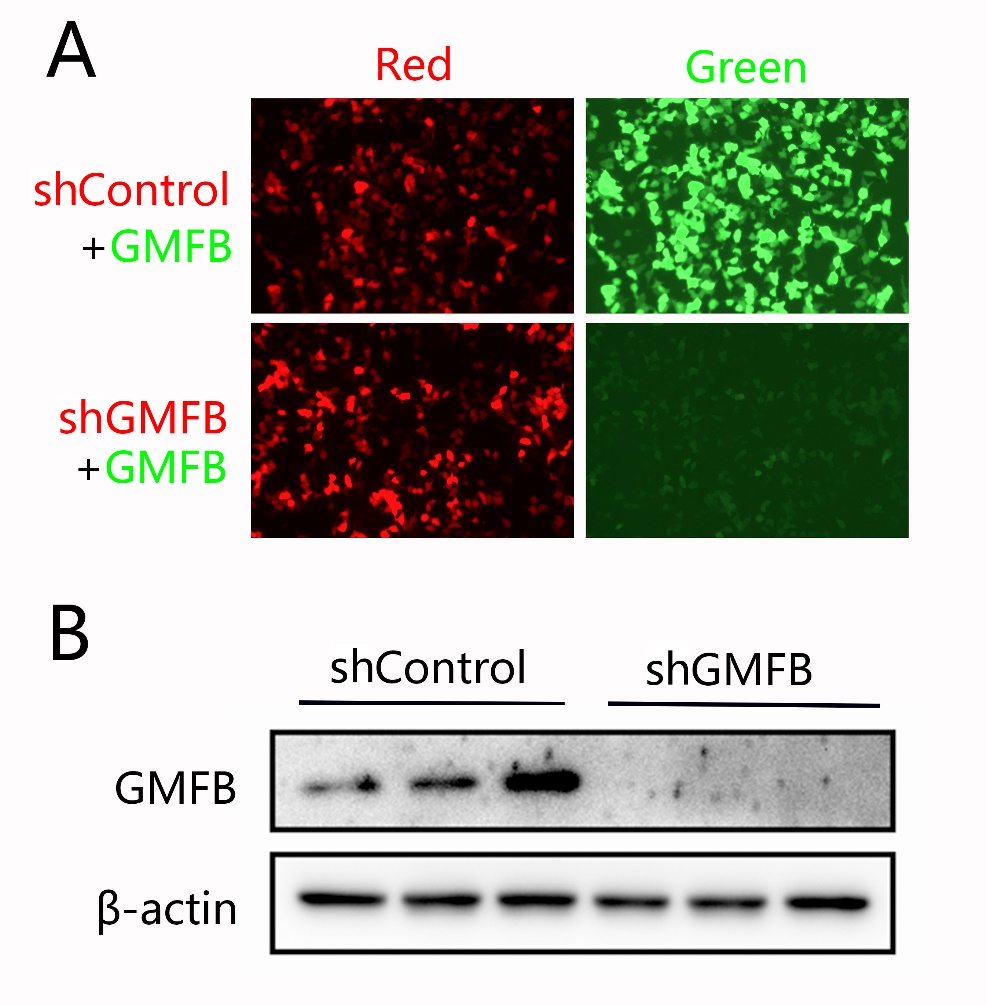


**Figure S13. shRNA inhibited GMFB expression in vitro and in vivo.**

(A) The snRNA (red) and GMFB (green) overexpression plasmid were used to co-transfect 293T cells in vitro and fluorescence attenuation tests indicated shRNA effectively inhibited GMFB expression.

(B) Western blotting analysis of GMFB expression in bone tissue from OVX rat received shRNA virus injection.

| **PMOP group** | **Control group** |
| --- | --- |
| (a) postmenopausal females aged 55-80 years at least 5 years since menopause with PMOP (mean lumbar 2-4 BMD T-score, ≦-2.5) | (a) non-postmenopausal females who had undergone spinal surgery for a lumbar degenerative disease (e.g., lumbar spondylolisthesis and lumbar spinal stenosis). |
| (b) acute fragile lumbar fractures in the prior 2 weeks and clear indication of vertebroplasty or internal fixation. | (b) no osteoporosis or other metabolism disease. |
| (c) normal levels of laboratory indicators. |  |
| (d) no intake of drugs affecting bone metabolism (e.g., corticosteroids, antacids containing aluminium, and heparin). |  |
| (e) no systemic disease affecting bone metabolism (e.g., secondary osteoporosis, osteogenesis imperfecta, diabetes). |  |
| (f) no osteoporotic fracture |  |
| (g) no severe liver or kidney dysfunction. |  |

**Table S1 Inclusion criteria for this study**

**Table S2. Primers for qRT-PCR in the experiment.**

| **Gene Symbol** | **Forward primer** | **Reverse primer** |
| --- | --- | --- |
| PPARγ | GAACGTGAAGCCCATCGAGG | GGAACACTTTGTCAGCGACTGG |
| C/EBPα | CGACTTCTACGAGGTGGAG | ATGTAGGCGCTGATGTCTAT |
| LPL | ACTTGTCCCACTCCGTATCTG | TATCAAGCTCCCAGCACTCA |
| PLIN1 | GAGAAGGGTGTACAGGGTGC | TCGGGTGTTGGCAGCATATT |
| RXRA | CCCATGGTCCCAAAAGATGC | CAACCAGAACAAGCCCCTCT |
| FABP4 | AAATGTGCGACGCCTTTGTG | CCAAGTCCCCTTCTACGCTG |
| ADIPOQ | CTGGCTCCAAGTGTATGGGG | TTTGATTCTCGGGGCTACGG |
| PLTP | CGTGCGTAGTTCTGTGGATG | CATCCTCTCGTCGTCATCCA |
| β-actin | ATCATGTTTGAGACCTTCAA | CATCTCTTGCTCGAAGTCCA |

**Table S3. Clinical characteristics of participants.**

|  | Osteoporosis  (n=11) | Control  (n=8) | P value |
| --- | --- | --- | --- |
| Age, year | 69.4±7.6 | 64.9±8.6 | >0.05 |
| Height, cm | 159±5 | 158±4 | >0.05 |
| Weight, kg | 57.2±7.2 | 63.9±8.2 | <0.05 |
| BMI, kg/m^2^ | 22.8±2.7 | 25.4±3.9 | >0.05 |
| T-scores in lumbar spine | -2.81±0.52 | -1.55±0.32 | <0.001 |
| T-scores in total hip | -2.55±0.47 | -1.19±0.59 | <0.001 |

Abb­reviations: BMI, body mass index

|  | NO. | age,  year | heigh,  cm | weight,  kg | BMI,  kg/m | T-scores in lumbar spine | T-scores in total hip | Glucose,  mmol/L | Cholesterol,  mmol/L | Triglyceride,  mmol/L | Thyroxine,  mmol/L | PTH,  pg/ml |
| --- | --- | --- | --- | --- | --- | --- | --- | --- | --- | --- | --- | --- |
| OP | 1 | 71 | 155 | 58 | 24.14 | -3.2 | -2.8 | 7.6 | 5.25 | 1.17 | 90.2 | 31.75 |
|  | 2 | 72 | 157 | 68 | 27.59 | -1.7 | -2.9 | 4.8 | 3.7 | 0.66 | 71.44 | 69.87 |
|  | 3 | 79 | 167 | 53 | 19.00 | -3.4 | -2.3 | 8.4 | 4.55 | 1.13 | 86.9 | 36.75 |
|  | 4 | 54 | 156 | 51 | 20.96 | -3.4 | -2.4 | 4.4 | 4.55 | 0.69 | 83.87 | 44.94 |
|  | 5 | 65 | 160 | 52 | 20.31 | -3.2 | -1.5 | 6.4 | 4.09 | 0.94 | 101 | 45.74 |
|  | 6 | 80 | 155 | 52 | 21.64 | -2.4 | -2.8 | 4.3 | 4.7 | 0.93 | 113.9 | 63.45 |
|  | 7 | 71 | 165 | 70 | 25.71 | -3.1 | -2.6 | 7 | 4.4 | 2.06 | 104.2 | 81.95 |
|  | 8 | 75 | 157 | 65 | 26.37 | -2.5 | -3 | 5.1 | 2.65 | 0.7 | 109.7 | 24.65 |
|  | 9 | 63 | 156 | 55 | 22.60 | -2.8 | -2 | 5.5 | 4.18 | 1.19 | 106 | 31.16 |
|  | 10 | 64 | 155 | 50 | 20.81 | -2.8 | -3.1 | 6.3 | 5.68 | 2.73 | 79.55 | 11.07 |
|  | 11 | 69 | 158 | 55 | 22.03 | -2.4 | -2.6 | 5.3 | 4.6 | 1.21 | 118.9 | 53.47 |
| Control | 1 | 53 | 158 | 62 | 24.84 | -1.6 | -1.6 | 5.9 | 4.23 | 1.45 | 76.28 | 51.64 |
|  | 2 | 71 | 162 | 45 | 17.15 | -1.6 | -0.9 | 4.2 | 3.9 | 1.8 | 70.91 | 48.68 |
|  | 3 | 77 | 156 | 63 | 25.89 | -1.7 | -1.5 | 6.7 | 5.12 | 1.05 | 83.02 | 48.34 |
|  | 4 | 67 | 160 | 65 | 25.39 | -1.8 | -0.3 | 5 | 4.27 | 1.06 | 90.01 | 50.17 |
|  | 5 | 57 | 166 | 69 | 25.04 | -1 | -0.8 | 5.1 | 5.75 | 1.62 | 108.9 | 36.46 |
|  | 6 | 71 | 150 | 70 | 31.11 | -1.8 | -1.8 | 5.1 | 5.6 | 1.31 | 116.2 | 47.24 |
|  | 7 | 65 | 159 | 70 | 27.69 | -1.8 | -0.7 | 5.1 | 4.25 | 0.82 | 77.44 | 54.21 |
|  | 8 | 58 | 161 | 67 | 25.85 | -1.1 | -1.9 | 4.5 | 4.13 | 1.74 | 80.55 | 20.14 |

**Table S4 The demographic data of patients**

**Table S4 (continued)**

|  | NO. | Cortisol,  ug/dl | Aldosterone,  pg/ml | Anti-O antibody,  U/ml | Rheumatoid factor,  IU/ml | Calcium,  mmol/L | Phosphorus,  mmol/L | β-CTX,  ng/ml | PINP,  ng/ml | (25-HO) Vit D,nmol/L | GMFB relative expression |
| --- | --- | --- | --- | --- | --- | --- | --- | --- | --- | --- | --- |
| OP | 1 | 9.348 | 140.17 | 25 | 20 | 2.21 | 1.16 | 22.34 | 0.732 | 66.91 | 1.262990299 |
|  | 2 | 1.037 | 63.42 | 25 | 20 | 2.18 | 1.1 | 20.11 | 0.725 | 15.78 | 1.395501235 |
|  | 3 | 2.343 | 84.74 | 25 | 20 | 2.12 | 1.05 | 12.93 | 0.543 | 23.35 | 1.411275415 |
|  | 4 | 13.164 | 78.44 | 25 | 20 | 2.23 | 1.37 | 24.89 | 0.787 | 33.08 | 1.257793074 |
|  | 5 | 5.612 | 75.66 | 31.7 | 20 | 2.14 | 1.54 | 60.46 | 2.23 | 12.86 | 2.436832749 |
|  | 6 | 13.553 | 73.76 | 25 | 20 | 2.23 | 0.98 | 14.94 | 0.264 | 26.63 | 2.384222829 |
|  | 7 | 23.638 | 180.5 | 72.5 | 20 | 2.23 | 1.15 | 13.39 | 0.464 | 24.42 | 2.304165541 |
|  | 8 | 26.274 | 130.89 | 25 | 23 | 2.33 | 1.28 | 10 | 0.646 | 35.13 | 1.725571719 |
|  | 9 | 16.014 | 112.45 | 25 | 20 | 2.47 | 1.27 | 12.75 | 0.637 | 36.31 | 3.066324104 |
|  | 10 | 17.315 | 109.08 | 64.8 | 20 | 2.57 | 1.52 | 21.75 | 0.446 | 45.20 | 1.880623333 |
|  | 11 | 13.014 | 105.11 | 38.3 | 20 | 2.32 | 1.13 | 43.05 | 1.09 | 34.06 | 1.408270825 |
| Control | 1 | 12.428 | 79.63 | 48.7 | 20 | 2.21 | 1.09 | 10.57 | 0.46 | 27.11 | 0.544241786 |
|  | 2 | 14.058 | 128.76 | 25 | 183 | 2.24 | 1.42 | 17.12 | 0.421 | 38.32 | 0.476026582 |
|  | 3 | 3.856 | 125.25 | 25 | 20 | 2.17 | 0.84 | 3.46 | 0.42 | 47.33 | 0.50353217 |
|  | 4 | 7.135 | 76.12 | 25 | 20 | 2.51 | 1.03 | 11.86 | 0.574 | 50.23 | 0.540979856 |
|  | 5 | 7.837 | 125.6 | 25 | 20 | 2.3 | 1.23 | 18.4 | 0.495 | 54.92 | 0.448357213 |
|  | 6 | 18.204 | 112.62 | 58 | 20 | 2.28 | 0.9 | 22.43 | 0.927 | 38.87 | 0.530854221 |
|  | 7 | 12.253 | 111.06 | 36.8 | 23.5 | 2.23 | 0.98 | 4.46 | 0.155 | 30.96 | 0.742374736 |
|  | 8 | 13.024 | 114.23 | 25 | 20 | 2.38 | 1.21 | 17.45 | 0.628 | 100.9 | 0.602986528 |
